# Supplementary material for: Spiral phyllotaxis predicts left-right asymmetric growth and style deflection in mirror-image flowers of Cyanella alba
Source: Nat Commun. 2025 Apr 18;16:3695. doi: 10.1038/s41467-025-58803-5 (PMC12008388; doi:10.1038/s41467-025-58803-5)
Supplement: Supplementary file 1 — Supplementary information [file 41467_2025_58803_MOESM1_ESM.pdf]

Supporting Information Text

Establishment of a reference genome assembly and sequencing of left- and right-biased individuals of *Cyanella alba* subsp*. flavescens*

To identify the presumed causal enantiostyly locus, we selected 25 *C. alba* individuals from sites in the Biedouw Valley, Western Cape, South Africa that had four or more open flowers of consistent handedness (15 left- and 10 right-handed plants, maximum flower number = 8; mean flower number = 5.2; Supp. Table S1) in September 2021. In addition, during the 2022 flowering season we selected 20 individuals (11 left- and 9 right-handed) which produced six or more flowers of consistent handedness (max = 12; mean = 7.5; Supp. Table S1) during a long-term monitoring study. Sixteen of the individuals from 2022 were derived from the same site in the Biedouw Valley as the plants sampled in 2021, while four plants were sampled from a site approximately 5 km North of the 2021 site. We subjected extracted DNA to individual Illumina whole-genome sequencing. Over 2 billion 150 bp paired end reads were produced across the 25 samples of the Illumina2021 dataset, with a median of 76.7 million reads produced per sample. Given an estimated haploid genome size of 0.98 Gb from flow cytometry, this represents roughly 23.5-fold coverage on average per sample. For the Illumina2022 set, sequencing of these 20 samples yielded an additional 1.6 billion 150 bp reads, with a median of 78.4 million reads produced per sample, representing roughly 24.3-fold coverage on average.

To facilitate the analysis of these sequences, we also established a reference genome assembly based on PacBio high-fidelity sequencing. For this we extracted DNA from an R-individual, C_209, which had formed seven consistently R-flowers in 2021. An R-morph was chosen, because an initial analysis of the Illumina2021 dataset using KmerGO2 (https://github.com/ChnMasterOG/KmerGO2) to identify morph-associated k-mers had indicated that in the 10 R-morph genomes the ratio of right- to left-associated k-mers was much higher than the converse ratio in the 15 L-morph genomes, suggesting that R-morphs are heterozygous for the causal locus and thus contain a high number of k-mers that are not found in L-morphs. Almost 21 Gb of reads were produced, which equates to a 21X genome coverage based on the flow cytometry genome size estimate, with a mean read length of 17,907 bp, and an N50 of 17,976 bp. Estimating the genome size based on k-mer frequencies using the counting tool KMC and the profiling tool GenomeScope2.0 resulted in an estimate of 700 Mb, thus smaller than the flow cytometry-based estimate of 0.98Gb. We used hifiasm to assemble two reference genomes, using either 1 Gb or 700 Mb as haploid genome sizes. BUSCO (Benchmarking Universal Single-Copy Orthologs) analysis indicated similarly high completeness for both assemblies. The “1 Gb” and “700 Mb” assemblies contain 2962 (91.6%)

and 2971 (91.9%) complete BUSCOs of the 3,236 BUSCO groups in the liliopsida_odb10 dataset. However, the former had a much higher percentage of duplicated BUSCOs (16.5%) than the latter (3.1%). Therefore, we continued our analysis with this latter assembly, which had a total length of 0.84 Gb (only slightly smaller than the flow cytometry-based estimate), and an N50 of 3.27 Mb.

Testing for a bi-allelic locus determining floral handedness

We tested two hypotheses regarding a possible simple, single-locus genetic basis of floral handedness in *C. alba*. The first was that one of the morphs is heterozygous and the other homozygous recessive. To test this, we performed a genome wide association study (GWAS) to identify single nucleotide polymorphisms (SNPs) associated with each phenotype. We aligned all 45 Illumina samples to the reference assembly and phenotype-associated SNPs were identified. The list of SNPs was filtered for sites where more than 80% of the individuals of one phenotype were heterozygous for the alternate alleles of the SNP, whereas in the other only <20% were heterozygous. We chose a relaxed threshold of 80% to account for the possibility that some individuals may have been mis-categorised, especially in the 2021 sample with its lower flower number per individual. In other words, we considered it possible that a genotypically L-plant might still have produced four R-flowers by a series of developmental errors or vice versa. The Illumina2021 and Illumina2022 datasets were first analysed separately, before combining all 45 samples (Table 1). In the Illumina2021 dataset, more heterozygous SNP sites were identified in R- than L-plants (Table 1), but the opposite pattern occurred in the Illumina2022 samples (Table 1). The combined dataset identified more SNPs that were heterozygous in L- than in R-plants. However, none of the identified SNPs were supported across more than one analysis. Therefore, we conclude that the identified SNPs are most likely unrelated to the putative *E* locus and that enantiostyly in *C. alba* does not appear to be governed by a bi-allelic Mendelian locus.

Testing for a hemizygous locus determining floral handedness

Our second hypothesis was that the putative *E* locus is hemizygous and present only in either L- or R-plants. As our reference genome was from a presumed R-plant, we first searched for an R-exclusive region by performing a coverage analysis. We calculated the number of Illumina reads mapping to each region of the haploid *C. alba* genome and compared left and right pools to identify regions where there was a significant difference in coverage. We used the two haploid assemblies generated by Hifiasm separately for this analysis. As above, we performed the analysis first with the Illumina2021 and Illumina2022 datasets separately, and then with the combined dataset of all 45 samples. Again, none of the windows identified as having significantly different coverage in L- compared to R-samples were shared between the

Illumina2021 and Illumina2022 datasets (Table S1), and no plausible candidate for a hemizygous region in the right-handed *C. alba* assembly was identified.

We next asked whether the putative *E* locus may be exclusively present in L-plants and thus undetectable with our R-plant reference assembly. Such a hemizygous region should be detectable based on L-plant exclusive *k*-mers in the L-pool. To search for L-morph exclusive k-mers, KmerGO2 version 2.01 was run on the Illumina2021 and Illumina2022 datasets sequentially, using the following settings: *k*-mer size: 40 bp, threads: 24 for kmc3 and filtering steps, eight threads for union step.

The Illumina2021 dataset showed the same result as obtained previously using 31-mers (Figure 1A). The number of right-associated *k*-mers (63,103) exceeds the number of left-associated *k*-mers (26,856). Two left-handed individuals (C_002 and C_010) show an unexpectedly high number of right-associated *k*-mers compared to other L-morphs; however, they still possess far fewer right-associated *k*-mers than the ten right-handed plants, and also possess more left-associated *k*-mers than all the R-morph plants, making it unlikely that they represent R-morph plants mis-categorised as L-morphs. In contrast with the Illumina2021 dataset, analysis of the Illumina2022 samples identified 443,456 left-, but only 73,343 right-associated *k*-mers. Surprisingly, all individuals, regardless of handedness, possessed more left- than right-associated *k*-mers. The much higher number of morph-associated kmers in the Illumina2022 than the Illumina2021 dataset likely reflects the smaller sample size of the former (20 as opposed to 25 individuals), and thus possibly higher rate of false-positive morph-associated k-mers. Among those morph-associated kmers, only 103 were shared by both datasets. Among them, 13 are left-associated in both datasets, 2 are right-associated in both datasets, 12 are left-associated in the Illumina2021 dataset but are right-associated in the Illumina 2022 dataset, and 76 are right-associated in the Illumina2021 dataset but are left-associated in the Illumina 2022 dataset. Since there is no agreement between the Illumina2021 and Illumina2022 datasets in this analysis, there is no strong support for a hemizygous region exclusive to left-handed plants.

Testing for transcripts exclusively expressed in plants with one floral handedness

As a complement to the coverage- or *k*-mer based search for a hemizygous region, we used RNA-seq of dissected styles (including the upper half of the ovary) to search for morph-specific transcripts. We harvested triplicate samples of styles from very early-, early- and mid-stage buds of plants with exclusively R- or L-flowers (see Supporting Information Text for definition of stages). No style deflection is visible in very early-stage buds, but this becomes detectable in early buds and obvious in mid-stage buds (see Figure 3A below). Thus, our samples should cover the critical time window when the orientation of style deflection is determined. We

identified 137 differentially expressed genes between L- and R-samples (FDR: $\alpha < 0.05$), of which 52 were upregulated and 85 downregulated in R- versus L-buds (Table S2). None of the genes were exclusively expressed in L- or R-plants. Thus, the transcriptomic analysis does not provide evidence for genes only present or only expressed in one of the phenotypes. Taken together, our results show no convincing evidence for a simple genetic polymorphism determining floral handedness in *C. alba*.

Biomechanical model

To describe the mechanical forces at play during ovary expansion, and to make predictions on what area in the ovary walls drive the expansion of the organ as a whole, we propose a two-fold apporach: first, based on the images of six ovaries of Cyanella alba flavescens (FigureS3A), we model them as polyhedron that best represent their morphological characteristics. This is achieved by considering as modelling polyhedron a skewed triangular prism topped by a truncated pyramid (Figures S4). We use a data-driven approach by approximating the two dimensional images of C. alba flavescens to the best fitting two dimensional projection of our polyhedron according to length, area, and perimeter of the image (Figure S5). Subsequently, under the hypothesis that differential wall expansion in C. alba flavescens results from the expansion of a carpel with the others being pulled by elastic coupling forces acting at a cellular level, we fit such a resulting polyhedron with another polyhedron with springs as edges and massless beads as nodes (Figure 3F). With numerical optimisation techniques like random search algorithms, we obtain the biomechanical parameters of the bead-spring system (i.e., the stiffness and elongation of the springs in the system) necessary for the latter to best reproduce the best fitting polyhedron identified by our data-driven approach (Figure S3B). The pipeline executing the analysis and parameter optimisation is written with Wolfram Mathematica, see our Mathematica notebook (Supplemental File lateR1.nb) for detail.

Data-driven model: best fitting polyhedron to expanded ovary

A three-dimensional polyhedron to model *C. alba flavescens* ovaries

Based on images of *C. alba flavescens*, we modelled the ovaries at the early stage of development - i.e., before the direction of stylar deflection can be determined, as triangular prisms topped by truncated pyramids. These geometric solids effectively capture the main morphological features of the ovaries, including the base of the style, and the stylised curvature of the external walls (see Figure S4A,B). We made the reasonable assumption that the ovary has rotational symmetry of order 3 at this early developmental stage. Our analysis involved fitting the two-dimensional projection of an ovary from above (i.e., with vein 1 in the field-of-view) with the polyhedron that can best reproduce such a projection.

We labelled the nodes at the base of the style within the model polyhedron as α, β, and γ. The nodes where the ovary roughly ceases to be vertically straight relative to the base of the ovary and begins to curve were denoted as a, b, and c (see Figure S5A,B). We denoted the positions of the nodes of the polyhedron as $\mathbf{r}_j = (x_j, y_j, z_j)$, with $j = a, b, c, \alpha, \beta, \gamma$. In the microscopy images, nodes c and γ are not visible and, hence, their position could not be identified. Therefore, we assumed that $z_b = z_c$ and $z_\beta = z_\gamma$. This means that, in our models, carpel 2 and 3 were equivalent both before and after expansion. We set the base of the ovary at height $z = 0$. The outer edges of the solid trace the midveins of the three carpels, see Figure 3B for a section of the ovary overlapped with the top view of our model.

We denoted the length of the edges connecting the base of the ovary to a, b, and c as $l_a = z_a$, $l_b = z_b$, $l_c = z_c$, respectively. Instead, we define the following lengths: $l_t = |\mathbf{r}_a - \mathbf{r}_b| = |\mathbf{r}_a - \mathbf{r}_c| = |\mathbf{r}_b - \mathbf{r}_c|$, $l_s = |\mathbf{r}_\alpha - \mathbf{r}_\beta| = |\mathbf{r}_\alpha - \mathbf{r}_\gamma| = |\mathbf{r}_\beta - \mathbf{r}_\gamma|$, and $l_u = |\mathbf{r}_a - \mathbf{r}_\alpha| = |\mathbf{r}_b - \mathbf{r}_\beta| = |\mathbf{r}_c - \mathbf{r}_\gamma|$. Note that

$$l_t = \frac{2}{\sqrt{3}}|x_a - x_b|. \tag{S1}$$

$$l_s = \frac{2}{\sqrt{3}}|x_\alpha - x_\beta|, \tag{S2}$$

$$l_u = \sqrt{(z_a - z_\alpha)^2 + \left(\frac{l_t - l_s}{\sqrt{3}}\right)^2}. \tag{S3}$$

Finally, though not part of the polyhedron, we denoted the shortest length of the segments connecting nodes α, β, and γ with the base of the ovary as $l_\alpha = z_\alpha$, $l_\beta = z_\beta$, and $l_\gamma = z_\gamma$, see Figure S4A for a schematic representation.

Morphological features of expanded ovaries

We used the same solid to model ovaries in the late stage of development, except in the latter case the solid was skewed. We experimentally observe that stylar bending is driven by differential carpel elongation (see Result section in the main text), with the arbitrarily denominated carpel 1 being the most elongating one. The inner and outer walls of carpel 1 are, without loss of generality from now on considered as those connecting the base of the ovary with nodes a and α. We made the assumption that expansion of carpels 2 and 3 is comparable. Therefore, we maintained the assumption that $z_b = z_c$ and $z_\beta = z_\gamma$ even after ovary expansion.

Morphological features of six images of the lateral view of ovaries of *C. alba flavescens* were measured with Wolfram Mathematica, see our Mathematica notebook (Supplemental File lateR1.nb) for details. The six images (Figure S3) consist of two ovaries with left-handed symmetry (labelled by L1 and L2) and three with right-handed symmetry (R1, R2, and R3) at late developmental stage, and one right-handed ovary at mid developmental stage (R4). Specifically, we measured the distance between the base of the ovary and the base of the style, i.e., $\overline{z}_\alpha$ and $\overline{z}_\beta$, the perimeter of the ovary $\overline{P}$, its area $\overline{A}$, the length of the ovary base $|\overline{x}_a - \overline{x}_b|$, and the length of the style base $|\overline{\mathbf{r}}_\alpha - \overline{\mathbf{r}}_\beta|$, see Figure S5C and D. Measured quantities are listed in Table S2.

Table S2: Morphological measurements. All numerical values except for $\overline{A}$ are expressed in millimeters. $\overline{A}$ is expressed in millimeters squared.

Ovary ID	$\overline{z}_\alpha$	$\overline{z}_\beta$	$\overline{P}$	$\overline{A}$	$\lvert\overline{x}_a - \overline{x}_b\rvert$	$\lvert\overline{\mathbf{r}}_\alpha - \overline{\mathbf{r}}_\beta\rvert$
L1	5.3	4.7	14.0	11.6	2.5	0.8
L2	4.1	3.9	11.9	8.8	2.4	0.7
R1	4.1	3.7	12.1	9.7	2.8	0.6
R2	4.2	3.6	12.4	9.8	2.4	0.7
R3	3.3	2.8	9.5	6.0	2.0	0.6
R4	2.2	2.0	6.5	2.7	1.4	0.5

Two-dimensional projection and fitting

We projected the expanded polyhedron defined before on the vertical plane passing through the nodes a and α and perpendicular to the edges that connect b and c, see Figure S4C,D. With simple trigonometry, it is possible to show that the equation for such a projection is

$$\begin{aligned} T(x) = &\left[z_a + \frac{(z_\alpha - z_a)}{\frac{l_t - l_s}{\sqrt{3}}} x\right] \Theta\left(\frac{l_t - l_s}{\sqrt{3}} - x\right) \\ &+ \left[z_\alpha + (z_\beta - z_\alpha)\frac{x - \frac{l_t - l_s}{\sqrt{3}}}{\frac{\sqrt{3}}{2} l_s}\right] \Theta\left(-\frac{l_t - l_s}{\sqrt{3}} + x\right) \Theta\left(\frac{l_t - l_s}{\sqrt{3}} + \frac{\sqrt{3}}{2} l_s - x\right) \\ &+ \left[z_\beta + (z_b - z_\beta)\frac{x - \frac{l_t - l_s}{\sqrt{3}} - \frac{\sqrt{3}}{2} l_s}{l_t - \frac{l_t - l_s}{\sqrt{3}} - \frac{\sqrt{3}}{2} l_s}\right] \Theta\left(-\frac{l_t - l_s}{\sqrt{3}} - \frac{\sqrt{3}}{2} l_s + x\right) \Theta\left(\frac{\sqrt{3}}{2} l_t - x\right), \end{aligned} \tag{S4}$$

where $\Theta(\cdot)$ is the Heaviside theta function. The area A of the projected polyhedron can be calculated using the previous equation as functions of z_a, z_b, z_α, and z_β:

$$A(z_a, z_b, z_\alpha, z_\beta) = \int_0^\infty \mathrm{d}x\, T(x), \tag{S5}$$

The perimeter of the projected polyhedron, instead, is

$$P(z_a, z_b, z_\alpha, z_\beta) = z_a + |\mathbf{r}_a - \mathbf{r}_\alpha| + |\mathbf{r}_\alpha - \mathbf{r}_\beta| + |\mathbf{r}_\beta - \mathbf{r}_b| + z_b + |x_a - z_b|. \tag{S6}$$

This projection of the polyhedron wass then used to fit the two dimensional images of *C. alba flavescens* through optimisation of the model quantities $z_a, z_b, z_\alpha, z_\beta$ to best reproduce the morphological parameters of the six analysed images of *C. alba flavescens* according to the target function

$$f_D(z_a, z_b, z_\alpha, z_\beta) = (z_\alpha - \overline{z}_\alpha)^2 + (z_\beta - \overline{z}_\beta)^2 + \left(P(z_a, z_b, z_\alpha, z_\beta) - \overline{P}\right)^2 + \left(A(z_a, z_b, z_\alpha, z_\beta) - \overline{A}\right)^2. \tag{S7}$$

We minimised this function by Nelder Mead method (Nelder & Mead, 1965) of the function NMinimize of Wolfram Mathematica to obtain the optimal values for $z_a, z_b, z_\alpha, z_\beta$, and we denoted them as z_j^{min}. Such optimal values are listed in Table S3. We found a very small mean relative squared error (MRSE) for each of the geometrical optimisation, i.e., for the six ovaries analysed MRSE$< 10^{-3}$.

Table S3: Numerical values for best fitting polyhedra. Numerical values are expressed in millimeters.

Ovary ID	z_a^{min}	z_α^{min}	z_β^{min}	z_b^{min}
L1	4.1	5.3	3.2	4.7
L2	3.3	4.1	1.0	3.4
R1	2.9	4.1	2.6	3.7
R2	3.7	4.2	2.6	3.6
R3	2.3	3.3	2.0	2.8
R4	1.5	2.2	1.5	2.0

Biomechanical model: a bead-spring system to reproduce ovary expansion

In this section, we modelled the family of polyhedra presented in the previous section as systems of beads and springs, with springs representing the edges and beads representing the nodes

of a polyhedron (Figure 3F). With our bead-spring system, we are interested in reproducing the polyhedra identified in the previous section as the best fitting to the expanded ovaries of *C. alba flavescens*. As such, we are interested in a static configuration of such a bead-spring system, rather than in an oscillatory behaviour.

Initial conditions before ovary expansion

In principle, to model the expansion of an ovary, it is necessary to observe both the initial morphology at an early stage of development, i.e., before stylar handedness could be determined, and the final morphology, after stylar deflection and differential ovary expansion have occurred. More specifically, it is necessary to know the equilibrium length of the springs of the system at the beginning of the expansion process. Under the assumption that ovary expansion only occurs along the z-axis, morphological features such as length of the style base $|\mathbf{r}_\alpha - \mathbf{r}_\beta|$, length of the base of an ovary $|x_a - x_b|$, and their derived quantities l_t and l_s can be considered constant throughout the expansion process, and we used the values reported in Table S2. Similarly, as we assumed that expansion only occurs in the first carpel, l_b, l_β, and l_u can be considered constant. Finally, due to the assumption of rotational symmetry of order 3, it follows that, before ovary expansion begins, $l_a = l_b$ and $l_\alpha = l_\beta$.

Unfortunately, we could only analyze the morphological features of one ovary at early stage development (Figure S5A,B), which we used to infer l_b and l_β (and, therefore, their depending quantity l_u) of the expanded ovaries. Based on the analysis of Figure S5A and B, we measured five morphological characteristics: the distances between base of the ovary and nodes a, α, β, and b (z_a^{early}, z_b^{early}, z_α^{early}, and z_β^{early}, respectively), and the length of the ovary base ($|x_a^{\text{early}} - x_b^{\text{early}}|$). The morphological features are listed in Table S4. Then, we defined the equilibrium length of the

Table S4: Morphological measurements of the ovary in Figure S5A,B at early stage development. Numerical values are expressed in millimeters.

z_a^{early}	z_b^{early}	z_α^{early}	z_β^{early}	$\lvert x_a^{\text{early}} - x_b^{\text{early}}\rvert$
0.7	0.5	1.6	1.6	1.3

springs a and b of the ovary in Figure S5A,B as the average between the two length measurements, i.e., $l_b = (\overline{z}_a + \overline{z}_b)/2 = 0.6$mm. Similarly, we defined $l_\beta = (\overline{z}_\alpha + \overline{z}_\beta)/2 = 1.6$mm.

We used the length of the ovary base of the images of expanded ovaries (Figure S3) in relation

to the same quantity in the ovary at an early stage (Figure S5A,B) to infer l_b and l_β for the expanded ovaries. To do so, we defined the scaling factor

$$\text{Scaling factor} = \frac{|x_a - x_b|}{|x_a^{\text{early}} - x_b^{\text{early}}|}, \tag{S8}$$

to re-scale l_b and l_β according to the following equations:

$$l_b = \frac{z_a^{\text{early}} + z_b^{\text{early}}}{2} \frac{|x_a - x_b|}{|x_a^{\text{early}} - x_b^{\text{early}}|}, \tag{S9a}$$

$$l_\beta = \frac{z_\alpha^{\text{early}} + z_\beta^{\text{early}}}{2} \frac{|x_a - x_b|}{|x_a^{\text{early}} - x_b^{\text{early}}|}. \tag{S9b}$$

The scaling factors are listed in Table S5.

Table S5: Scaling factors

Ovary ID	L1	L2	R1	R2	R3	R4
Scaling factor	2.0	2.1	2.2	2.1	1.7	1.1

Bead-spring system

Before expansion, the system consisted of a regular prism with triangular basis topped by a truncated pyramid. The vertices of this polyhedron are represented by beads of mass m, whereas the edges are replaced by spring with equilibrium length l_j, with $j = a, b, c, \alpha, \beta, \gamma$, and spring constant as follows. We made the assumption that corresponding walls of different carpels possessed the same stiffness. Therefore, external springs, corresponding to springs denoted by a, b, c, have spring constant k_e, which is also the elastic constant of spring connecting a to α, b to β, and c to γ. Inner springs, i.e., springs denoted by α, β, γ have elastic constant k_i. Springs connecting a, b, c have elastic constant k_u, whereas springs connecting α, β, γ - i.e., the base of the style, have elastic constant k_s.

Dynamic equations

Typically, the static equilibrium configuration $\mathbf{z}^{\text{eq}} = (z_a^{\text{eq}}, z_b^{\text{eq}}, z_c^{\text{eq}}, z_\alpha^{\text{eq}}, z_\beta^{\text{eq}}, z_\gamma^{\text{eq}})$ of positions of the beads consists in minimizing the potential energy of the system. In our case, directly solving for the equilibrium configuration was computationally demanding. Therefore, we tackled this challenge by solving the dynamic equations controlling the system. Through iteration of the dynamic solution, we identified an approximate static solution, thereby increasing the computational efficiency.

The dynamics of the bead-spring system is described by a system of second order linear ODEs. Given that our goal was to find the equilibrium configuration $\mathbf{z}^{\text{eq}}$, we assumed, without loss of generality, that all beads in the system have identical mass. Consequently, we treat the elastic constants, denoted by k, as per unit mass. This assumption is justified as our model neglects gravitational and non-conservative forces, indicating that the equilibrium configuration - defined as the configuration that minimizes potential energy, is independent of the mass of the beads. Then, the dynamics equations are

$$\begin{cases} \ddot{\mathbf{r}}_a = -k_e(\mathbf{r}_a - l_a\mathbf{v}) - k_t(\mathbf{r}_a - \mathbf{r}_b - \mathbf{l}_{ab}) - k_t(\mathbf{r}_a - \mathbf{r}_c - \mathbf{l}_{ac}) - k_u(\mathbf{r}_a - \mathbf{r}_\alpha - \mathbf{l}_{a\alpha}), \\ \ddot{\mathbf{r}}_b = -k_e(\mathbf{r}_b - l_b\mathbf{v}) - k_t(\mathbf{r}_b - \mathbf{r}_a - \mathbf{l}_{ba}) - k_t(\mathbf{r}_b - \mathbf{r}_c - \mathbf{l}_{bc}) - k_u(\mathbf{r}_b - \mathbf{r}_\beta - \mathbf{l}_{b\beta}), \\ \ddot{\mathbf{r}}_c = -k_e(\mathbf{r}_c - l_c\mathbf{v}) - k_t(\mathbf{r}_c - \mathbf{r}_a - \mathbf{l}_{ca}) - k_t(\mathbf{r}_c - \mathbf{r}_b - \mathbf{l}_{cb}) - k_u(\mathbf{r}_c - \mathbf{r}_\gamma - \mathbf{l}_{c\gamma}), \\ \ddot{\mathbf{r}}_\alpha = -k_i(\mathbf{r}_\alpha - l_\alpha\mathbf{v}) - k_s(\mathbf{r}_\alpha - \mathbf{r}_\beta - \mathbf{l}_{\alpha\beta}) - k_s(\mathbf{r}_\alpha - \mathbf{r}_\gamma - \mathbf{l}_{\alpha\gamma}) - k_u(\mathbf{r}_\alpha - \mathbf{r}_a - \mathbf{l}_{\alpha a}), \\ \ddot{\mathbf{r}}_\beta = -k_i(\mathbf{r}_\beta - l_\beta\mathbf{v}) - k_s(\mathbf{r}_\beta - \mathbf{r}_\alpha - \mathbf{l}_{\beta\alpha}) - k_s(\mathbf{r}_\beta - \mathbf{r}_\gamma - \mathbf{l}_{\beta\gamma}) - k_u(\mathbf{r}_\beta - \mathbf{r}_b - \mathbf{l}_{\beta b}), \\ \ddot{\mathbf{r}}_\gamma = -k_i(\mathbf{r}_\gamma - l_\gamma\mathbf{v}) - k_s(\mathbf{r}_\gamma - \mathbf{r}_\alpha - \mathbf{l}_{\gamma\alpha}) - k_s(\mathbf{r}_\gamma - \mathbf{r}_\beta - \mathbf{l}_{\gamma\beta}) - k_u(\mathbf{r}_\gamma - \mathbf{r}_c - \mathbf{l}_{\gamma c}), \end{cases} \tag{S10}$$

where $\mathbf{v} = (0, 0, 1)$ is a unit vector in the z-direction, and $\mathbf{l}_{ij}$ is a vector with origin in $\mathbf{r}_i$ and directed towards $\mathbf{r}_j$ with length l_{ij}. Note that, while the vector $\mathbf{l}_{ij}$ depends on $\mathbf{r}_i$ and $\mathbf{r}_j$, the initial length l_{ij}^0 of the corresponding springs is

$$l_{ij}^0 = \begin{cases} l_t & \text{if} \quad i, j = a, b, c, \\ l_s & \text{if} \quad i, j = \alpha, \beta, \gamma, \\ l_u & \text{if} \quad (i, j) = (a, \alpha), (b, \beta), (c, \gamma). \end{cases} \tag{S11}$$

Given that the nodes were constrained to oscillate in the z-direction, our system of 18 differential

equations reduced to 6 differential equation for the z-component only of the position vector **r** of the nodes. With simple trigonometry, it is possible to show that

$$(\ddot{\mathbf{r}}_j)_z = \ddot{z}_j, \tag{S12a}$$

$$(\mathbf{r}_j - l_j\mathbf{v})_z = z_j - l_j, \tag{S12b}$$

$$(\mathbf{r}_j - \mathbf{r}_m - \mathbf{l}_{jm})_z = \begin{cases} (z_j - z_m)\left(1 - \frac{l_t}{\sqrt{l_t^2 + (z_j - z_m)^2}}\right) & \text{if} \quad j, m = a, b, c, \\ (z_j - z_m)\left(1 - \frac{l_s}{\sqrt{l_s^2 + (z_j - z_m)^2}}\right) & \text{if} \quad j, m = \alpha, \beta, \gamma, \\ (z_j - z_m)\left(1 - \frac{l_u}{\sqrt{\frac{(l_t - l_s)^2}{3} + (z_j - z_m)^2}}\right) & \text{if} \quad (j, m) = (a, \alpha), (b, \beta), (c, \gamma). \end{cases} \tag{S12c}$$

As we were interested in the static solution, we used the following initial conditions for every bead j:

$$\begin{cases} \ddot{z}_j = 0, \\ \dot{z}_j = 0. \end{cases} \tag{S13}$$

Static solution

We were interested in finding the equilibrium configuration of the system, i.e., the initial conditions $z_j(t = 0) = z_j^0 = z_j^{\text{eq}}$ such that $\dot{z}_j(t)|_{z_j^{\text{eq}}} = 0$ and $\ddot{z}_j(t)|_{z_j^{\text{eq}}} = 0$ at every time t. We proceeded by iterations, i.e., we subsequently solved the system of Equations (S10) with initial conditions $z_j^0[n] = z_j^{\text{eq}}[n-1]$ and $\dot{z}_j^0[n] = 0$, where n indicates the nth iteration of the solving process, and where $z_j^0 = l_j$. We defined the equilibrium position of the node j as

$$z_j^{\text{eq}} = \lim_{n\to\infty} z_j^{\text{eq}}[n]. \tag{S14}$$

The solutions of Equations (S10) are oscillatory (Figure S6A) and, as such, $z_j[n] = z_j[n](t)$. Therefore, we defined the equilibrium conditions as the time average of such quantities, i.e.,

$$z_j^{\text{eq}}[n] = \lim_{T\to\infty} \frac{1}{T}\int_0^T \mathrm{d}t\, z_j[n](t). \tag{S15}$$

In our analyses, we used a more practical approach by integrating between $t = 0$ and $t = 200$

sec. Indeed, this time integration period is sufficiently long to provide a reliable approximation of the time average while remaining short enough to allow for the numerical computation of the solution of the system of ODE within a reasonable timeframe. We considered z_j to be at their equilibrium after the iteration $n = N$ if, for every $j = a, b, c, \alpha, \beta, \gamma$,

$$\frac{\left| z_j^{\text{eq}}[N] - z_j^{\text{eq}}[N-1] \right|}{z_j^{\text{eq}}[N-1]} < 0.0001. \tag{S16}$$

Then, in our analysis, we approximated $z_j^{\text{eq}} \simeq z_j^{\text{eq}}[N]$. To validate this approximation, we plotted the dynamic solution of the system of Equations S10 for the six ovaries analysed in this study, using $z_j^0 = z_j^{\text{eq}}$. As shown in Figure S6B, our iterative method provides an accurate approximation of the solution of the system.

Optimisation: linking mechanical and data-driven models

We used the procedure as described in the previous section to find the polyhedron resulting from bead-spring model under static solution that best reproduced the polyhedron as identified by our data-driven modelling in the first section. To do so, we minimized the function f_M defined by

$$f_M\left(l_a^{\text{opt}}, l_\alpha^{\text{opt}}, l_\beta^{\text{opt}}, l_b^{\text{opt}}, k_e^{\text{opt}}, k_i^{\text{opt}}, k_s^{\text{opt}}, k_t^{\text{opt}}\right) = \left(z_a^{\text{eq}} - z_a^{\text{min}}\right)^2 + \left(z_b^{\text{eq}} - z_b^{\text{min}}\right)^2 + \left(z_\alpha^{\text{eq}} - z_\alpha^{\text{min}}\right)^2 + \left(z_\beta^{\text{eq}} - z_\beta^{\text{min}}\right)^2. \tag{S17}$$

Parameters z_j^{eq} are actually functions of the dynamic parameters of the models, i.e., l_a, l_b, l_α, l_β, and k_e, k_i, k_s, k_t, k_u. In sticking with the assumption that only l_a and l_α can elongate unconstrained with respect of the initial length of early stage ovaries, we allowed for a maximum elongation of l_b and l_β of 10% respect to their initial length.

In our optimization, we used the measurements reported in Table S3 for z_j^{min} and their derived quantities l_s, l_t, and l_u. Instead, l_b and l_β have been inferred according to the procedure described in the section "Initial conditions before ovary expansion" and the re-scaling factors listed in Table S5.

We performed parameter optimisation with the function NMinimize in Mathematica, by using the Nelder-Mead controlled random search algorithm. The results of our parameter optimisation are listed in Table S6. We used such parameters to reconstruct, through our mechanistic model and Equation (S4), the equilibrium configuration of the bead-spring system that best reproduced the

best fitting polyhedron after geometrical optimisation. Figure S3B and Table S7 show, respectively, the resulting fitting polyhedron, and a summary of the change in morphological characteristics of the six ovaries of *Cyanella alba flavescens* that we analysed.

Table S6: Numerical values for optimised parameters of the biomechanical model. Lengths are measured in millimeters, spring constants in Newton per millimeter per kilogram, while the mean relative squared error (MRSE) of the fit after optimisation is non-dimensional.

Ovary ID	l_a^{opt}	l_α^{opt}	l_β^{opt}	l_b^{opt}	k_e^{opt}	k_i^{opt}	k_s^{opt}	k_t^{opt}	MRSE
L1	5.6	6.3	3.5	1.2	7.3	1.3	16.2	9.8	0.04
L2	3.8	4.1	3.2	1.2	9.5	9.8	16.9	1.1	0.02
R1	3.1	4.1	3.3	1.5	9.3	1.0	15.6	2.1	0.04
R2	4.9	4.8	3.1	1.4	3.3	8.5	18.8	8.9	0.03
R3	2.9	3.5	2.6	1.2	9.8	10.0	19.2	9.8	0.03
R4	1.7	2.2	0.8	1.8	8.2	1.9	16.7	3.3	0.05

Table S7: Elongation of polyhedron edges compared to equilibrium length of the respective representing spring in our biomechanical models. Positive values correspond to edges which are shorter than the respective equilibrium length and are, therefore, compressed. Similarly, negative values correspond to edges that are longer than the respective equilibrium length and are, therefore, stretched. Numerical values are expressed in millimeters.

Ovary ID	$l_a^{\text{opt}} - z_a$	$l_\alpha^{\text{opt}} - z_\alpha$	$l_\beta^{\text{opt}} - z_\beta$	$l_b^{\text{opt}} - z_b$
L1	1.2	1.1	-0.4	-0.8
L2	0.5	0.1	-0.2	-0.1
R1	0.3	0.1	-0.3	-0.2
R2	1.4	0.5	-0.4	-0.4
R3	0.4	0.2	-0.2	-0.1
R4	0.2	0.0	-0.1	-0.1

Table S6 shows that our model consistently predicts a high stiffness for the base of the style k_s^{opt}. To check whether such a high stiffness is in fact necessary for the correct bending, we run our system of Equations S10 for each ovary with the optimised parameters as in Table S6, except for the stiffness at the base of the style, which we set at $k_s^{\text{low}} = k_s^{\text{opt}}/10$. Table S8 shows how the angle of stylar bending to the vertical axis of the ovary changes compared to the optimised case.

Table S8: Angle of the base of the style compared to the vertical axis as resulting from Equations S10 with optimised stiffness, and low stiffness.

Ovary ID	angle (optimised stiffness)	angle (low stiffness)
L1	$40.4°$	$62.3°$
L2	$35.0°$	$49.2°$
R1	$34.1°$	$53.8°$
R2	$35.5°$	$51.1°$
R3	$32.6°$	$51.5°$
R4	$29.2°$	$44.3°$

Supporting Information Figures

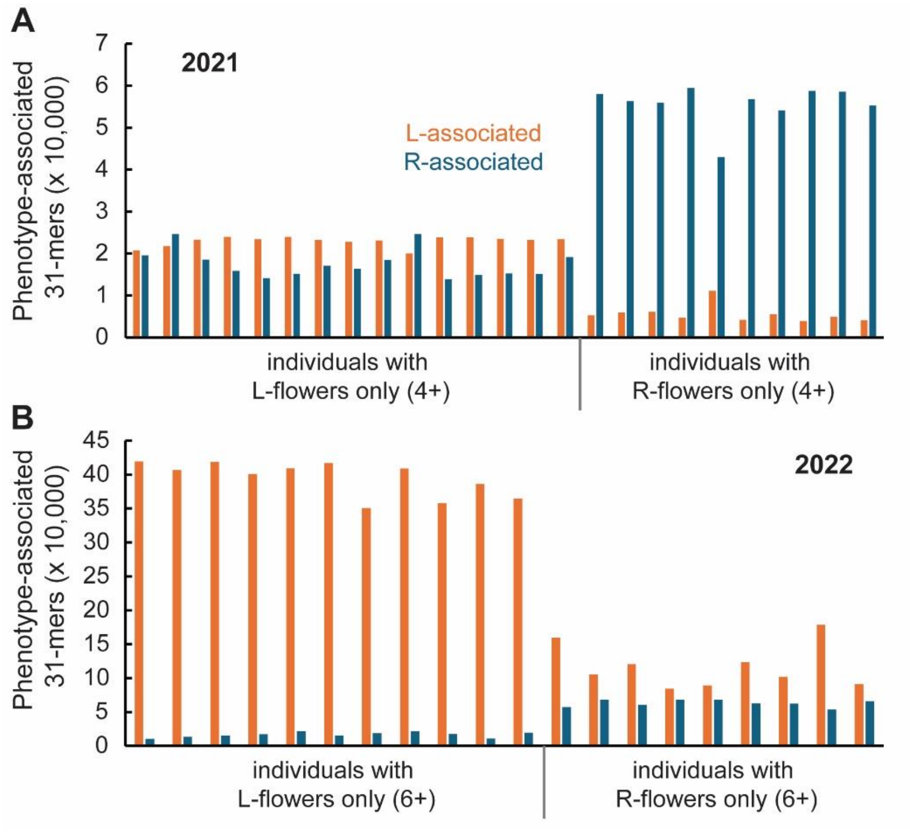

Figure S1: Lack of evidence for a genomic region associated with style orientation

(**A**,**B**) Phenotype-associated *k*-mer composition of *C. alba* individuals in the Illumina2021 (**A**) and Illumina2022 (**B**) datasets. *K*-mers associated with either the L- or R-pools were identified using KmerGO2. The total number of L- and R-associated 31-mers in each individual was calculated and plotted.

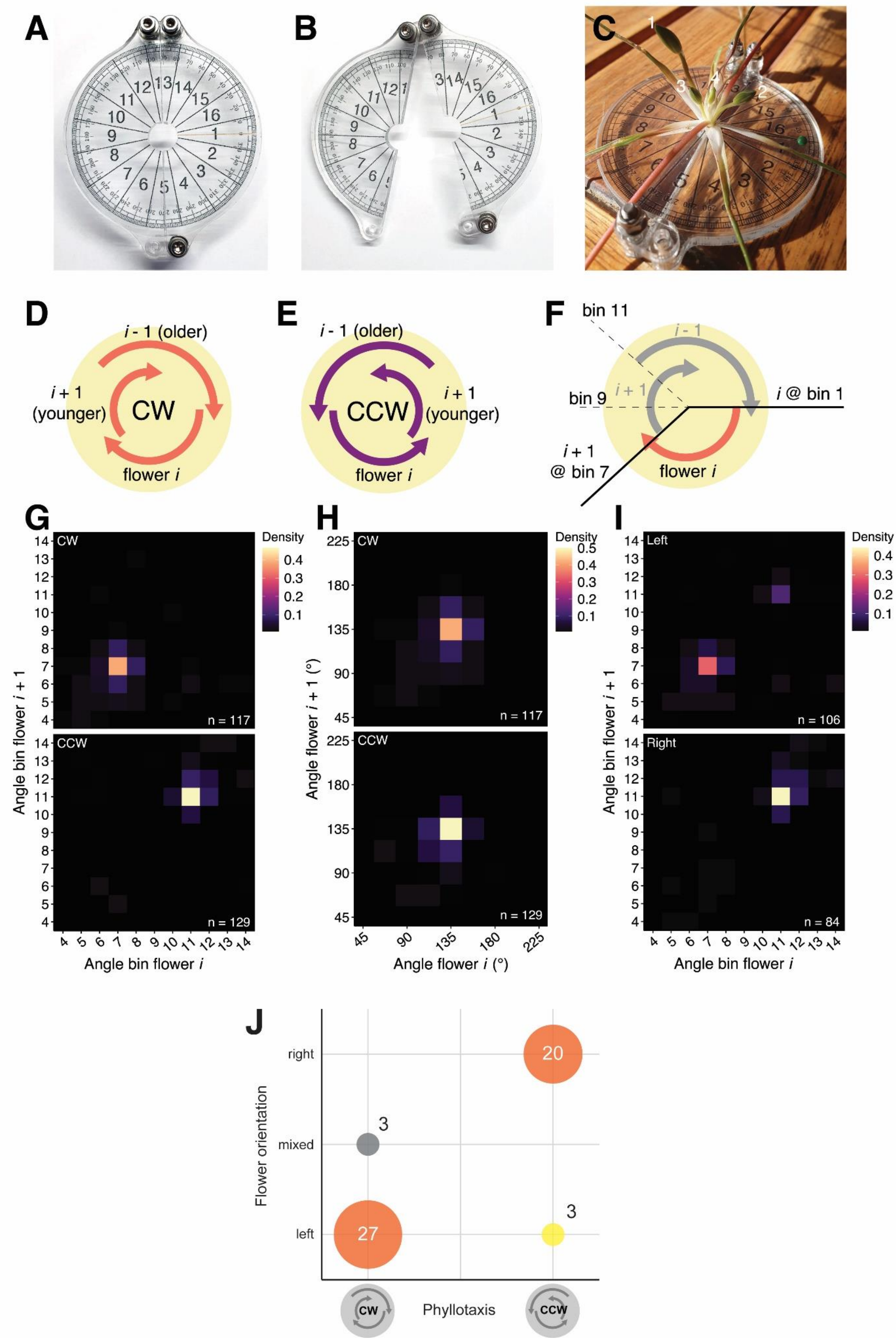

Figure S2: *Cyanella alba* exhibits spiral phyllotaxis.

(**A**, **B**) Overview of the hinged protractor for phyllotactic measurements in closed (**A**) and open (**B**) conformation. Multiple diameters of the central hole were produced and used, as a snug fit provides the most accurate measurements.

(**C**) Protractor around a *C. alba* plant exhibiting CW spiral phyllotaxis. Flower buds are labelled 1-4 (oldest to youngest). Two stalks of open flowers are also visible.

(**D**, **E**) Schematic diagram of three flowers on a plant with CW (**D**) or CCW (**E**) phyllotaxis.

(**F**) Visualization of phyllotactic measurements of a flower i in relation to the next (younger) flower i + 1 on a plant with CW phyllotaxis. In this scenario, the assigned angle bin would be '7'.

(**G**) 2D heatmap of measured angle bins of flowers i against i + 1 for all analyzed *C. alba* plants, separated based on the chirality of their phyllotaxis. Colour-scaled density represents the proportion of angles per panel falling in a specific 2D bin.

(**H**) Translation of the bins in (**G**) to degrees.

(**I**) 2D heatmap of angle bins separated on plants based on their predominant stylar orientation (left or right).

(**J**) Correlation of phyllotactic direction (CW: clockwise, CCW: counter-clockwise) and floral handedness of plants analyzed in 2024. Numbers in the diagram indicate the number of individual plants. The determination of floral handedness is based on between 2 and 5 open flowers and sufficiently old buds per plant.

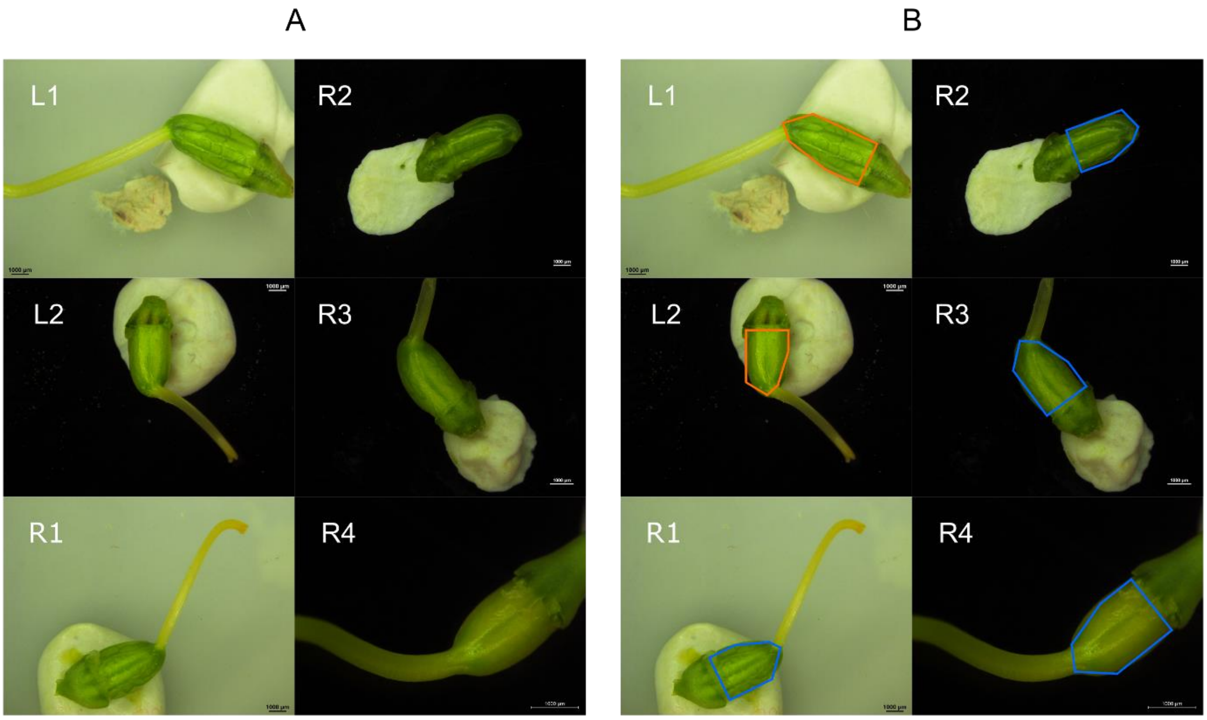

Figure S3: Starting images for gynoecium modelling.

(**A**) Lateral images of six ovaries of *C. alba flavescens*. (**B**) The same images are overlaid with the respective fitting polyhedron after parameter optimisation.

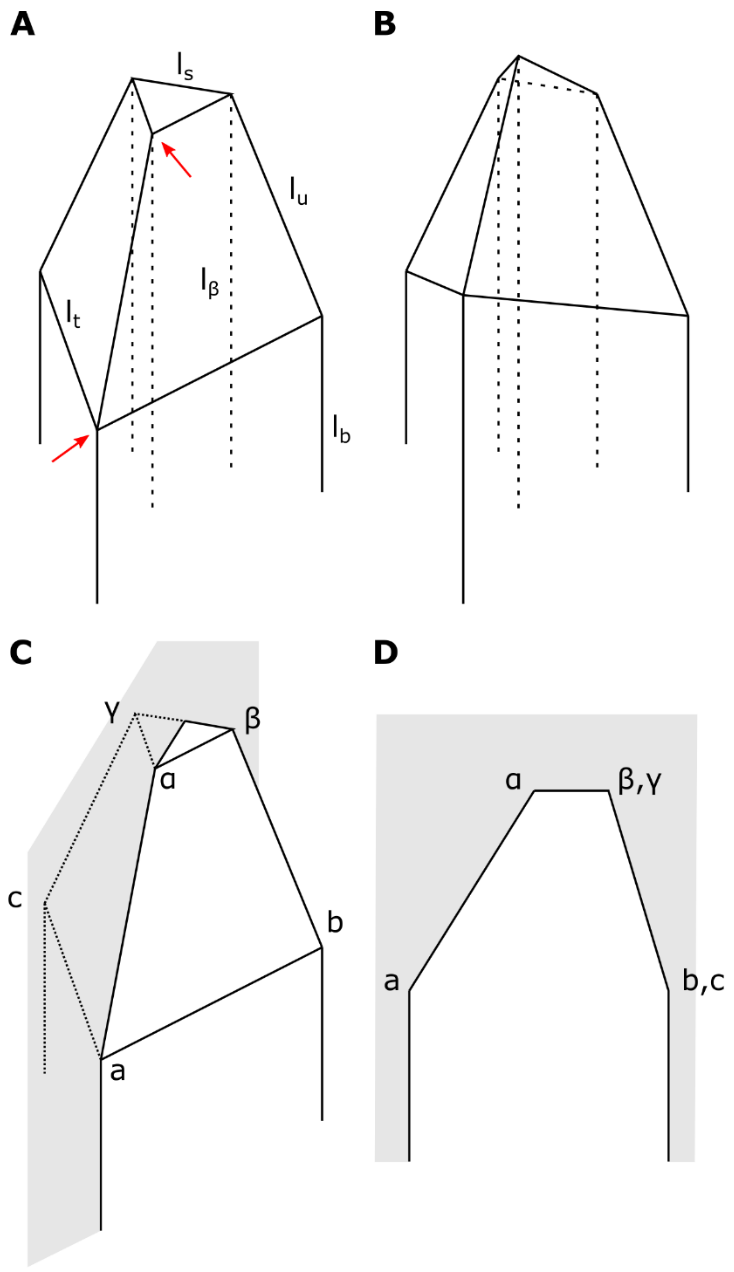

Figure S4: Modelling representation of the ovaries

(**A**) The ovaries are modelled as polyhedra composed of triangular prisms topped with a truncated pyramid. Red arrows denote the nodes corresponding to the top of the inner and outer walls of the expanding carpel.

(**B**) After expansion of the walls indicated by arrows in (**A**), a polyhedron appears skewed.

(**C**, **D**) Schematic of the plane over which the polyhedron is projected. The projection plane (in grey) passes through nodes *a* and *α*, and intersects the opposite edges forming a 90 degree angle (**C**). Lateral view of the projected polyhedron (**D**).

α, *β*, *γ* indicate the nodes at the base of the style, and *a*, *b*, *c* the nodes where the ovary roughly ceases to be vertically straight relative to the base of the ovary (**C**, **D**). l_a to l_γ indicate the shortest lengths of the segments connecting the respective nodes to the base of the ovary. Only l_b and l_β are shown for clarity.

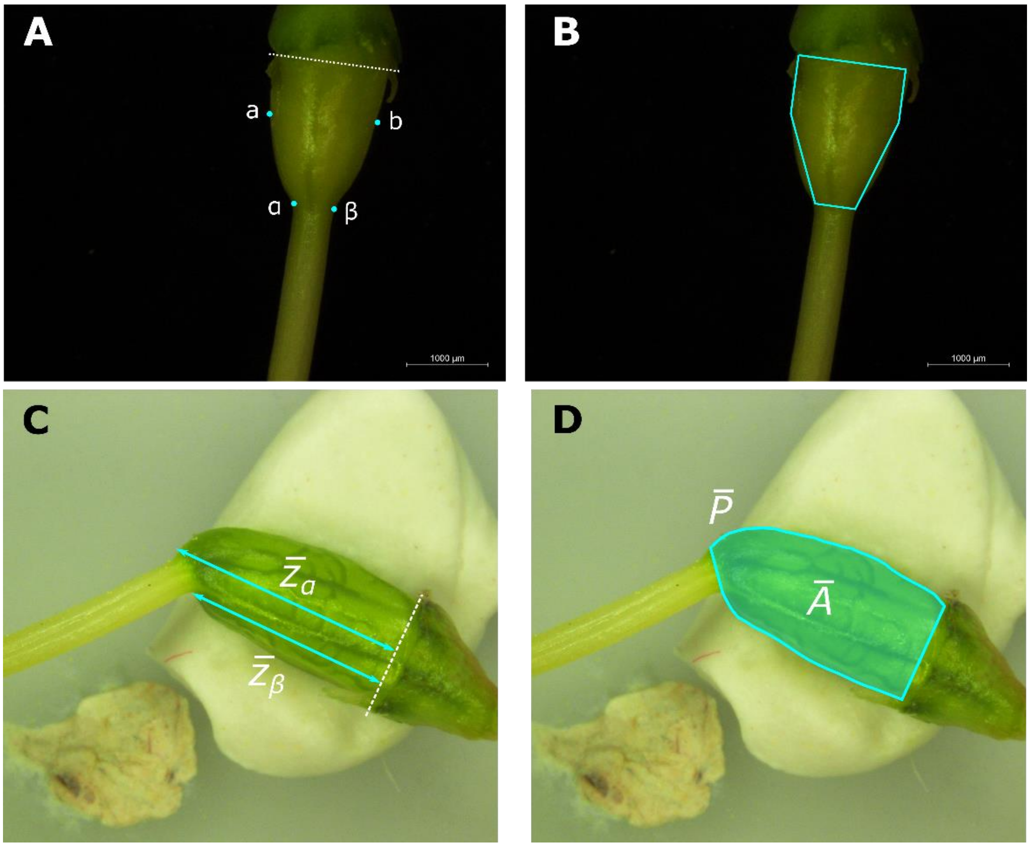

Figure S5: Morphology of ovaries at early and late stage of development.

(**A**, **B**) In this early-stage ovary, nodes *a* and *b* are identified as roughly the location when the ovary starts bending towards the style as observed from the microscopy image. The nodes *α* and β are at the base of the style (**A**). Based on *a*, *b*, *α* and β, the initial polyhedron is drawn (**B**).

(**C**, **D**) In this late-stage ovary, the blue lines represent the measure of the distance between the base of the ovary and the base of the style, at its extremes (**C**). The solid line represents the perimeter of the ovary, and the opaque area is used to measure the area of the ovary (**D**).

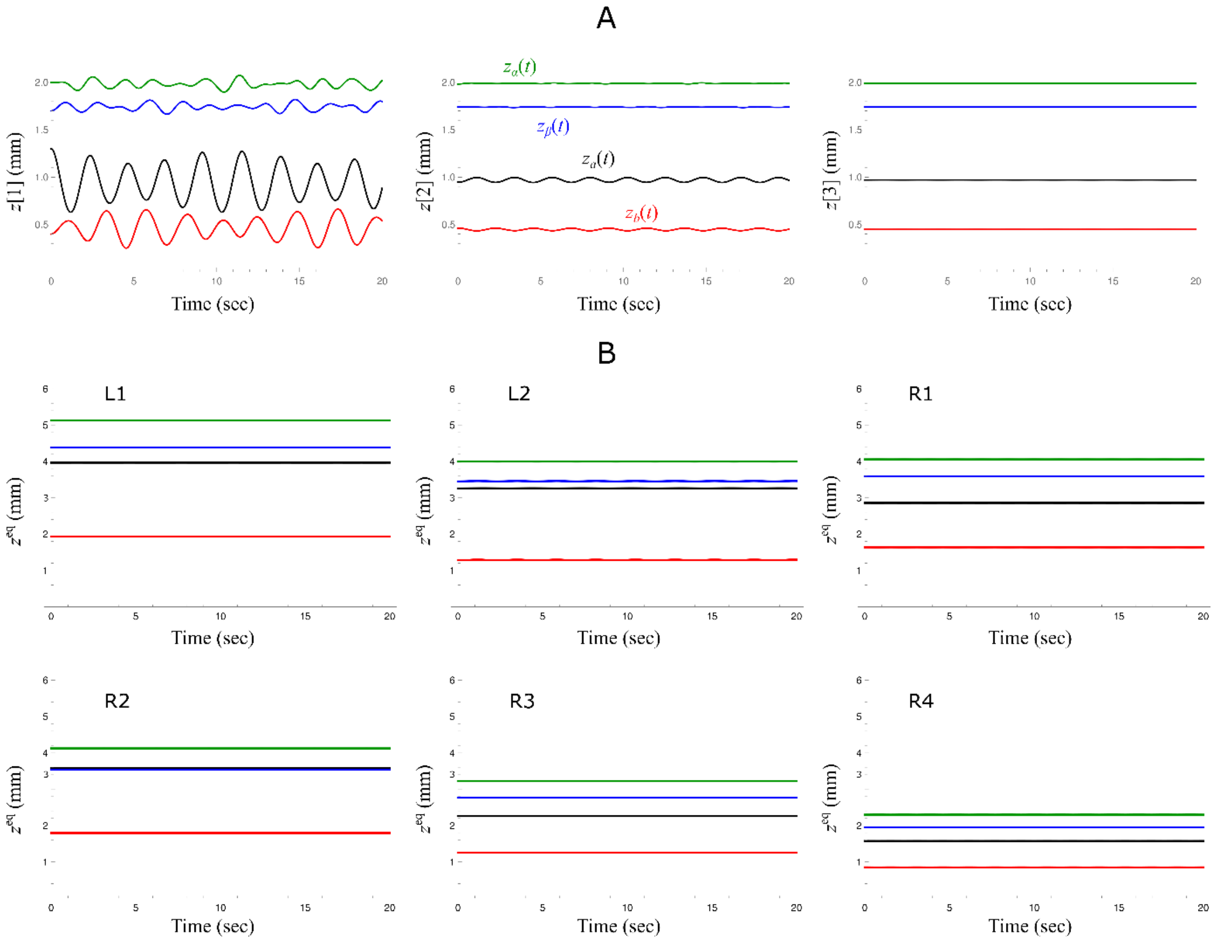

Figure S6: Solution of ODEs and equilibrium positions.

(**A**) Example of a solution of the system of ODEs Eqs. (S10) after the (from left to right) first, second, and third iteration for a generic set of initial conditions.

(**B**) Equilibrium solution of the system Eqs. (S10) for the six ovaries analysed, after the final iteration. In every plot from top to bottom, we show the solutions for (green) $z_\alpha(t)$, (blue) $z_\beta(t) = z_\gamma(t)$, (black) $z_a(t)$, and (red) $z_b(t) = z_c(t)$.

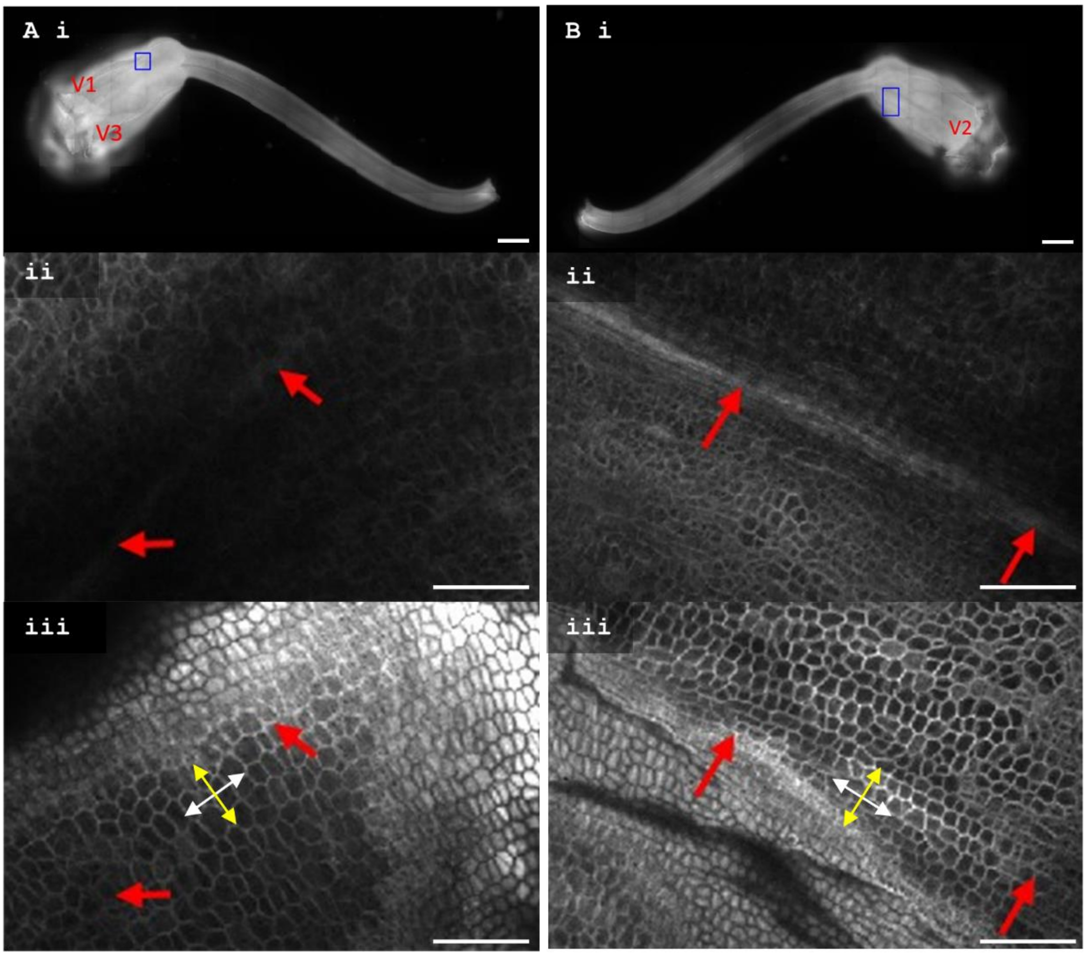

Figure S7: Illustration of cell length and width measurements in *Cyanella alba* subsp. *flavescens*

(**A**, **B**). A left-handed *C. alba* pistil stained with Calcoflour white is imaged from both sides Overviews (panels **i**) were generated using tile scanning (20% overlap) captured on a widefield Zeiss monochrome CCD camera. From view (**A**), veins 1 and 3 are visible (marked), whereas in view (**B**) vein 2 can be seen. Blue boxes indicate the approximate position of z-stacks **ii-iii**. The midveins (panels **ii**) are clearly visible just below the surface layer (panels **iii**) and are marked in the z-stack (red arrows). A line of 5-10 cells between the arrows (panels **iii**) were measured in ImageJ to calculate an average cell size measurement along vein 1 (**A**) and vein 2 (**B**). Cell length was measured in the direction indicated by the white arrows, whereas cell width was measured in the direction indicated by the yellow arrows. Scale bars in (**i**) represent 1000 µm, whereas in (**ii**) and (**iii**) they represent 100 µm.

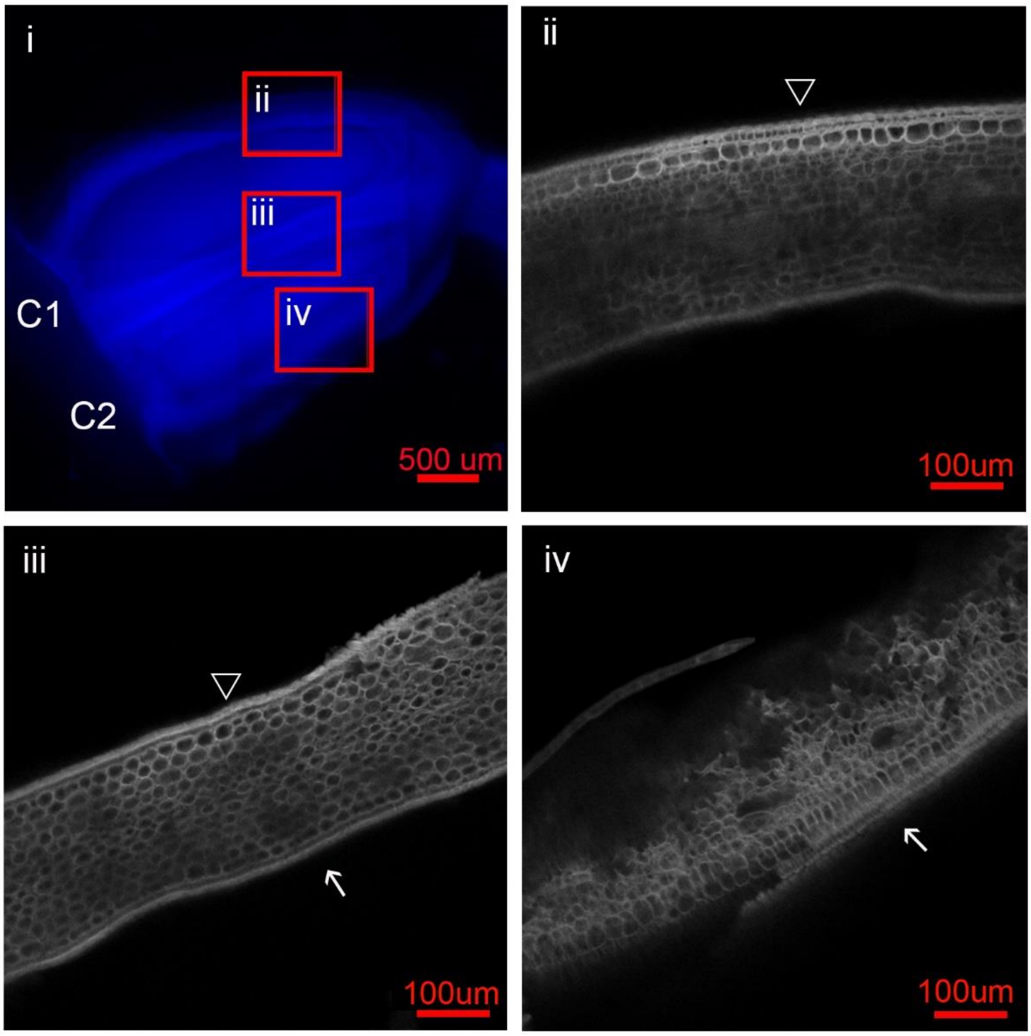

Figure S8: Illustration of measurements of cell lengths in outer and inner carpel walls

(**i**). A right-handed *C. alba* pistil was dissected, had its ovules removed, and stained with Calcoflour white. Red boxes indicate the approximate position of z-stacks. Selected images used for calculating cell length from these z-stacks are shown in ii-iv. A line of 10 subepidermal cells were measured in ImageJ to calculate the average cell size for the outer carpel walls (ii and iv), and inner carpel walls (iii) ∆ indicates walls of carpel 1, and the white arrow indicates the walls of carpel 2. Scale bar in (**i**) represents 500 µm, whereas in (**ii-iv**) they represent 100 µm.

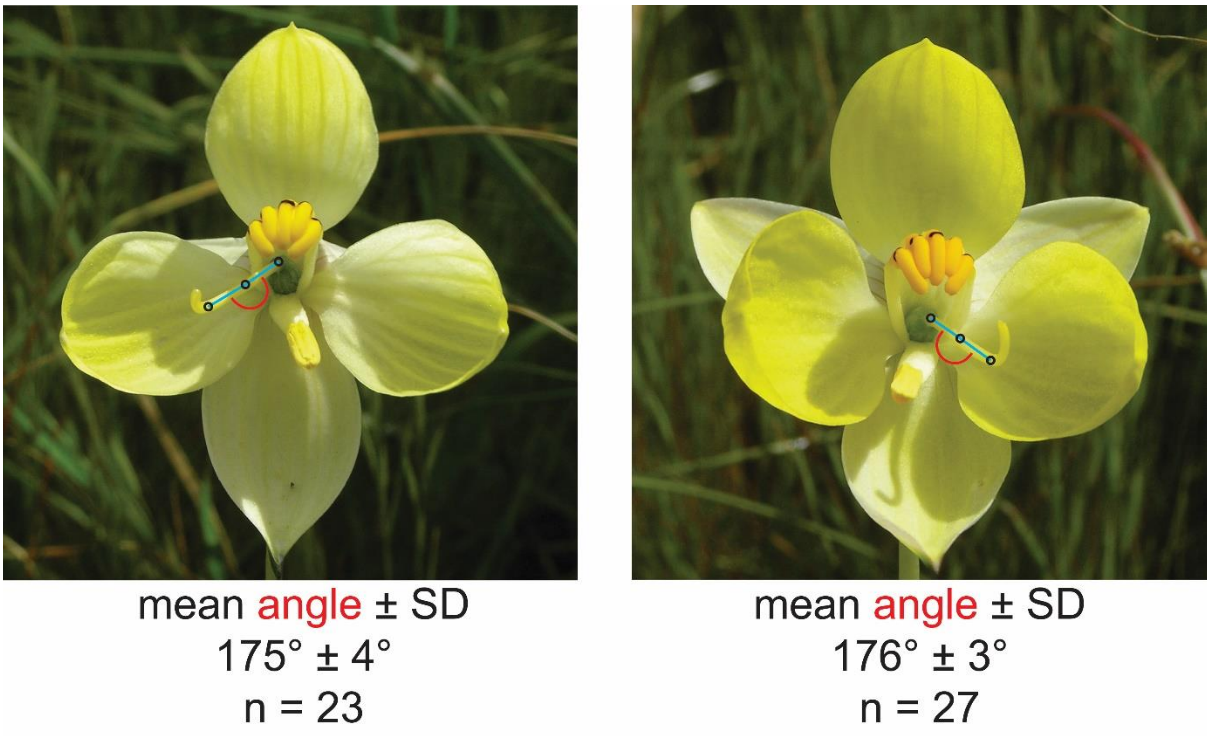

Figure S9: Styles themselves of *Cyanella alba* subsp*. flavescens* are essentially straight.

To determine whether the style itself is straight or curved, three points were defined at the style/ovary transition, the beginning of upward bend at the style tip, and the midpoint between these, as indicated by the black circles, and the angle (red) between the two cyan lines connecting these points was measured. Values underneath give the average angle ± SD and the number of styles measured.

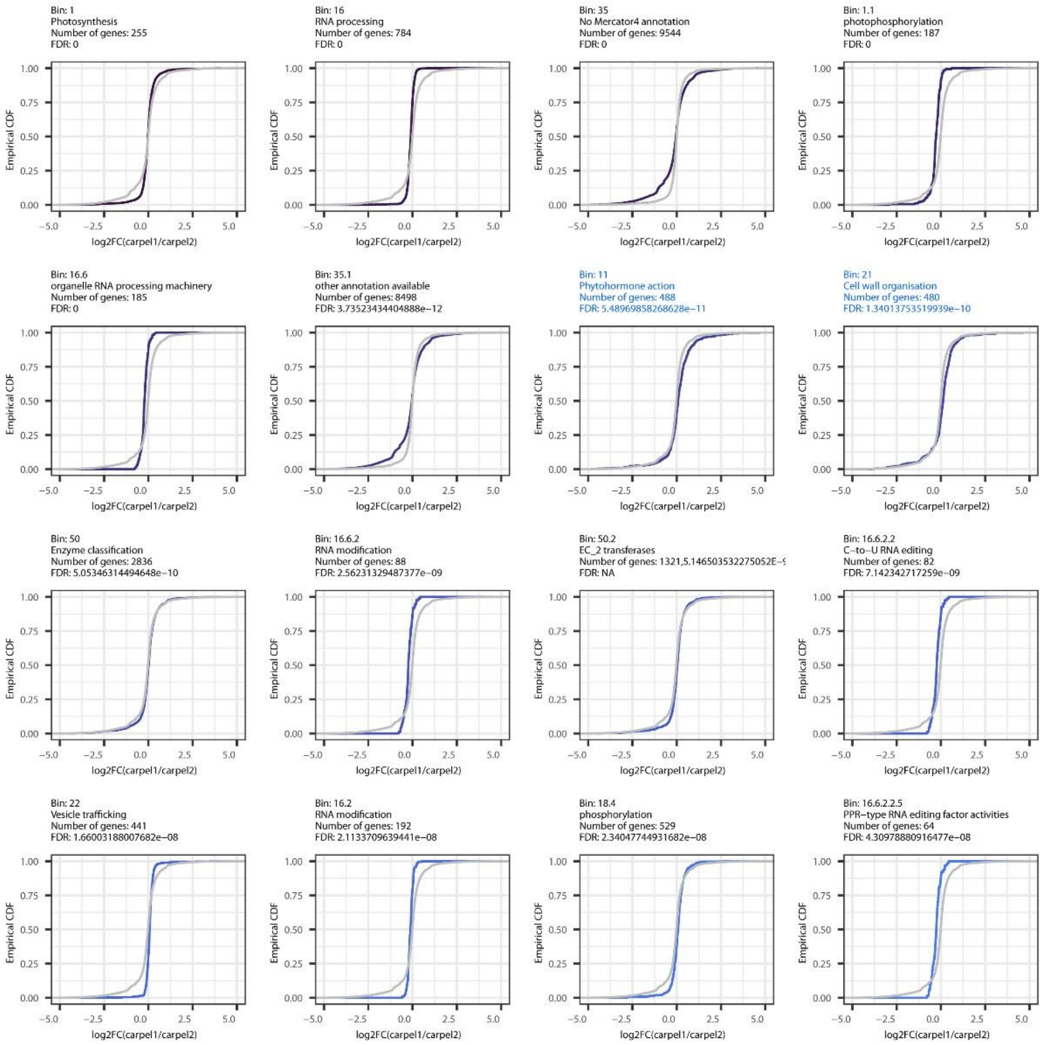
Bin: 1
Photosynthesis
Number of genes: 255
FDR: 0
Bin: 16
RNA processing
Number of genes: 784
FDR: 0
Bin: 35
No Mercator4 annotation
Number of genes: 9544
FDR: 0
Bin: 1.1
photophosphorylation
Number of genes: 187
FDR: 0
Bin: 16.6
organelle RNA processing machinery
Number of genes: 185
FDR: 0
Bin: 35.1
other annotation available
Number of genes: 8498
FDR: 3.73523434404888e−12
Bin: 11
Phytohormone action
Number of genes: 488
FDR: 5.48969858268628e−11
Bin: 21
Cell wall organisation
Number of genes: 480
FDR: 1.34013753519939e−10
Bin: 50
Enzyme classification
Number of genes: 2836
FDR: 5.05346314494648e−10
Bin: 16.6.2
RNA modification
Number of genes: 88
FDR: 2.56231329487377e−09
Bin: 50.2
EC_2 transferases
Number of genes: 1321,5.146503532275052E−
FDR: NA
Bin: 16.6.2.2
C−to−U RNA editing
Number of genes: 82
FDR: 7.142342717259e−09
Bin: 22
Vesicle trafficking
Number of genes: 441
FDR: 1.66003188007682e−08
Bin: 16.2
RNA modification
Number of genes: 192
FDR: 2.1133709639441e−08
Bin: 18.4
phosphorylation
Number of genes: 529
FDR: 2.34047744931682e−08
Bin: 16.6.2.2.5
PPR−type RNA editing factor activities
Number of genes: 64
FDR: 4.30978880916477e−08
Empirical CDF
log2FC(carpel1/carpel2)

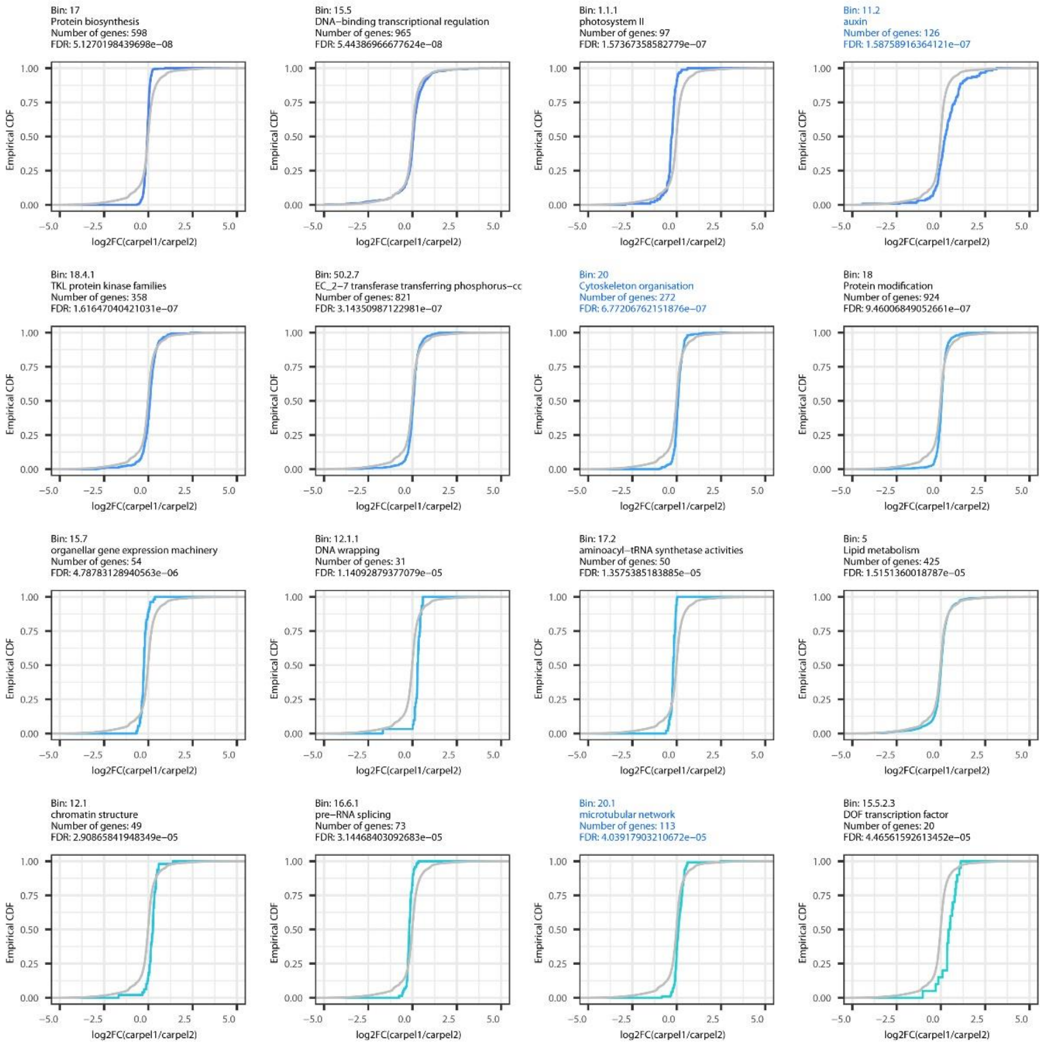
Bin: 17
Protein biosynthesis
Number of genes: 598
FDR: 5.1270198439698e−08
Bin: 15.5
DNA−binding transcriptional regulation
Number of genes: 965
FDR: 5.44386966677624e−08
Bin: 1.1.1
photosystem II
Number of genes: 97
FDR: 1.57367358582779e−07
Bin: 11.2
auxin
Number of genes: 126
FDR: 1.58758916364121e−07
Bin: 18.4.1
TKL protein kinase families
Number of genes: 358
FDR: 1.61647040421031e−07
Bin: 50.2.7
EC_2−7 transferase transferring phosphorus−cc
Number of genes: 821
FDR: 3.14350987122981e−07
Bin: 20
Cytoskeleton organisation
Number of genes: 272
FDR: 6.77206762151876e−07
Bin: 18
Protein modification
Number of genes: 924
FDR: 9.46006849052661e−07
Bin: 15.7
organellar gene expression machinery
Number of genes: 54
FDR: 4.78783128940563e−06
Bin: 12.1.1
DNA wrapping
Number of genes: 31
FDR: 1.14092879377079e−05
Bin: 17.2
aminoacyl−tRNA synthetase activities
Number of genes: 50
FDR: 1.3575385183885e−05
Bin: 5
Lipid metabolism
Number of genes: 425
FDR: 1.5151360018787e−05
Bin: 12.1
chromatin structure
Number of genes: 49
FDR: 2.90865841948349e−05
Bin: 16.6.1
pre−RNA splicing
Number of genes: 73
FDR: 3.14468403092683e−05
Bin: 20.1
microtubular network
Number of genes: 113
FDR: 4.03917903210672e−05
Bin: 15.5.2.3
DOF transcription factor
Number of genes: 20
FDR: 4.46561592613452e−05
Empirical CDF
log2FC(carpel1/carpel2)

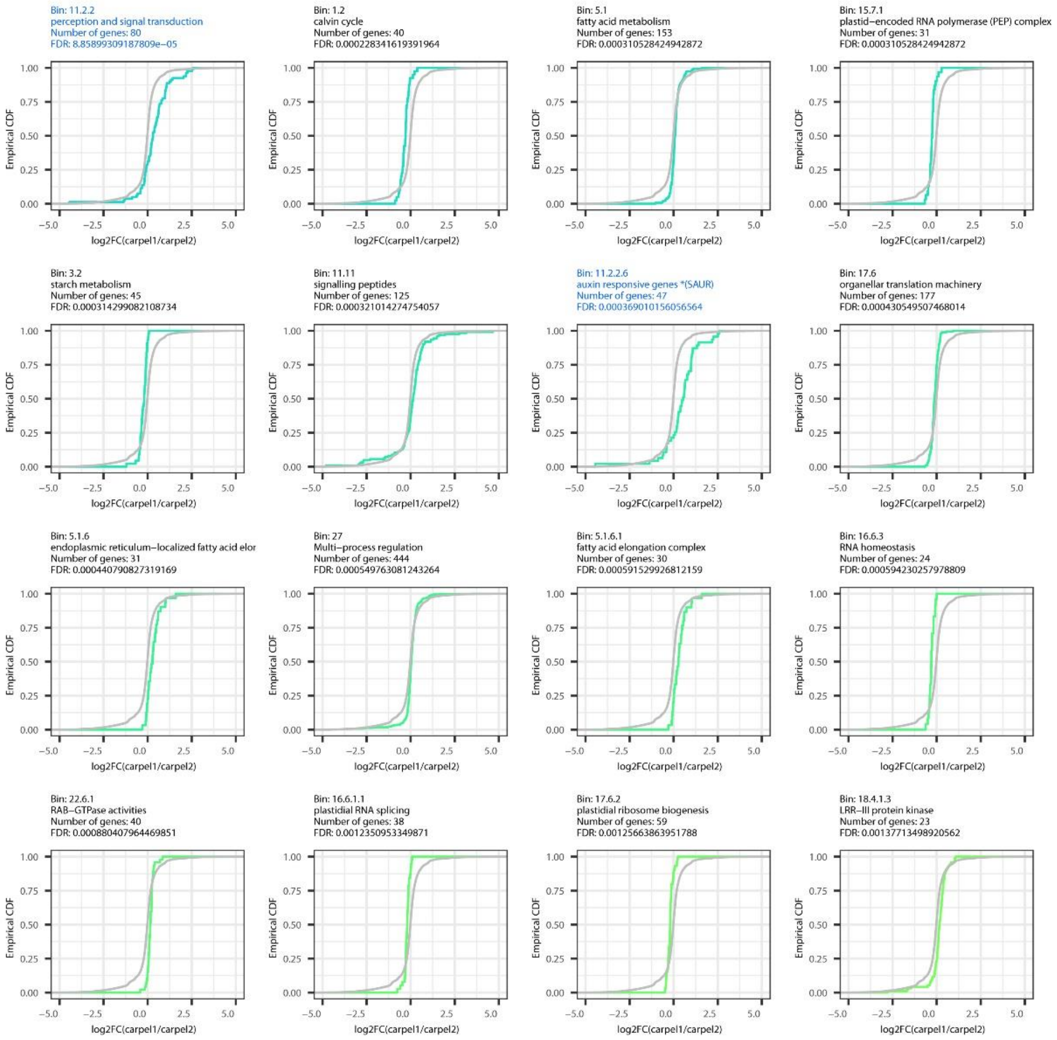

Bin: 11.2.2
perception and signal transduction
Number of genes: 80
FDR: 8.85899309187809e−05
Bin: 1.2
calvin cycle
Number of genes: 40
FDR: 0.000228341619391964
Bin: 5.1
fatty acid metabolism
Number of genes: 153
FDR: 0.000310528424942872
Bin: 15.7.1
plastid−encoded RNA polymerase (PEP) complex
Number of genes: 31
FDR: 0.000310528424942872
Bin: 3.2
starch metabolism
Number of genes: 45
FDR: 0.000314299082108734
Bin: 11.11
signalling peptides
Number of genes: 125
FDR: 0.000321014274754057
Bin: 11.2.2.6
auxin responsive genes *(SAUR)
Number of genes: 47
FDR: 0.000369010156056564
Bin: 17.6
organellar translation machinery
Number of genes: 177
FDR: 0.000430549507468014
Bin: 5.1.6
endoplasmic reticulum−localized fatty acid elor
Number of genes: 31
FDR: 0.000440790827319169
Bin: 27
Multi−process regulation
Number of genes: 444
FDR: 0.000549763081243264
Bin: 5.1.6.1
fatty acid elongation complex
Number of genes: 30
FDR: 0.000591529926812159
Bin: 16.6.3
RNA homeostasis
Number of genes: 24
FDR: 0.000594230257978809
Bin: 22.6.1
RAB−GTPase activities
Number of genes: 40
FDR: 0.000880407964469851
Bin: 16.6.1.1
plastidial RNA splicing
Number of genes: 38
FDR: 0.0012350953349871
Bin: 17.6.2
plastidial ribosome biogenesis
Number of genes: 59
FDR: 0.00125663863951788
Bin: 18.4.1.3
LRR−III protein kinase
Number of genes: 23
FDR: 0.00137713498920562
Empirical CDF
log2FC(carpel1/carpel2)

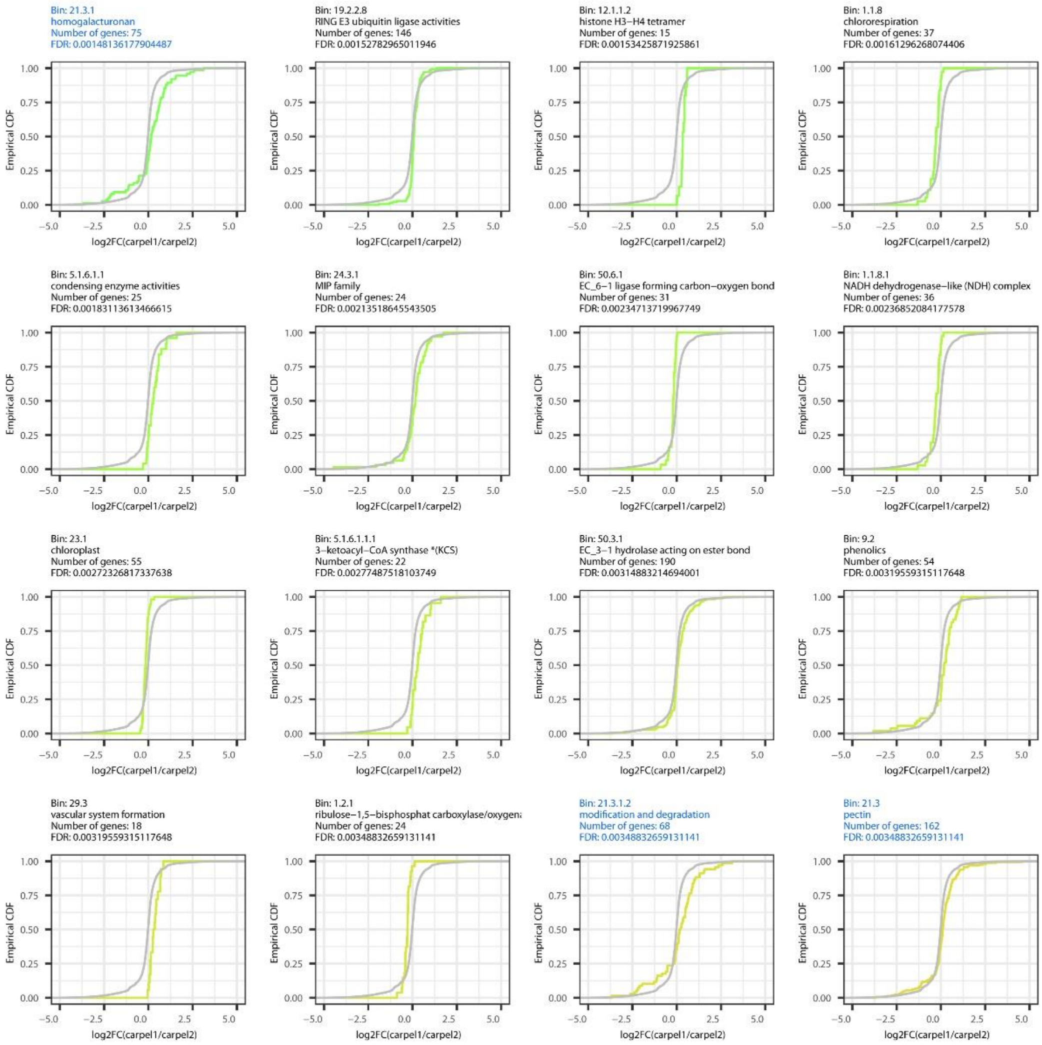
Bin: 21.3.1
homogalacturonan
Number of genes: 75
FDR: 0.00148136177904487
Bin: 19.2.2.8
RING E3 ubiquitin ligase activities
Number of genes: 146
FDR: 0.00152782965011946
Bin: 12.1.1.2
histone H3−H4 tetramer
Number of genes: 15
FDR: 0.00153425871925861
Bin: 1.1.8
chlororespiration
Number of genes: 37
FDR: 0.00161296268074406
Bin: 5.1.6.1.1
condensing enzyme activities
Number of genes: 25
FDR: 0.00183113613466615
Bin: 24.3.1
MIP family
Number of genes: 24
FDR: 0.00213518645543505
Bin: 50.6.1
EC_6−1 ligase forming carbon−oxygen bond
Number of genes: 31
FDR: 0.00234713719967749
Bin: 1.1.8.1
NADH dehydrogenase−like (NDH) complex
Number of genes: 36
FDR: 0.00236852084177578
Bin: 23.1
chloroplast
Number of genes: 55
FDR: 0.00272326817337638
Bin: 5.1.6.1.1.1
3−ketoacyl−CoA synthase *(KCS)
Number of genes: 22
FDR: 0.00277487518103749
Bin: 50.3.1
EC_3−1 hydrolase acting on ester bond
Number of genes: 190
FDR: 0.00314883214694001
Bin: 9.2
phenolics
Number of genes: 54
FDR: 0.00319559315117648
Bin: 29.3
vascular system formation
Number of genes: 18
FDR: 0.00319559315117648
Bin: 1.2.1
ribulose−1,5−bisphosphat carboxylase/oxygen
Number of genes: 24
FDR: 0.00348832659131141
Bin: 21.3.1.2
modification and degradation
Number of genes: 68
FDR: 0.00348832659131141
Bin: 21.3
pectin
Number of genes: 162
FDR: 0.00348832659131141
Empirical CDF
log2FC(carpel1/carpel2)

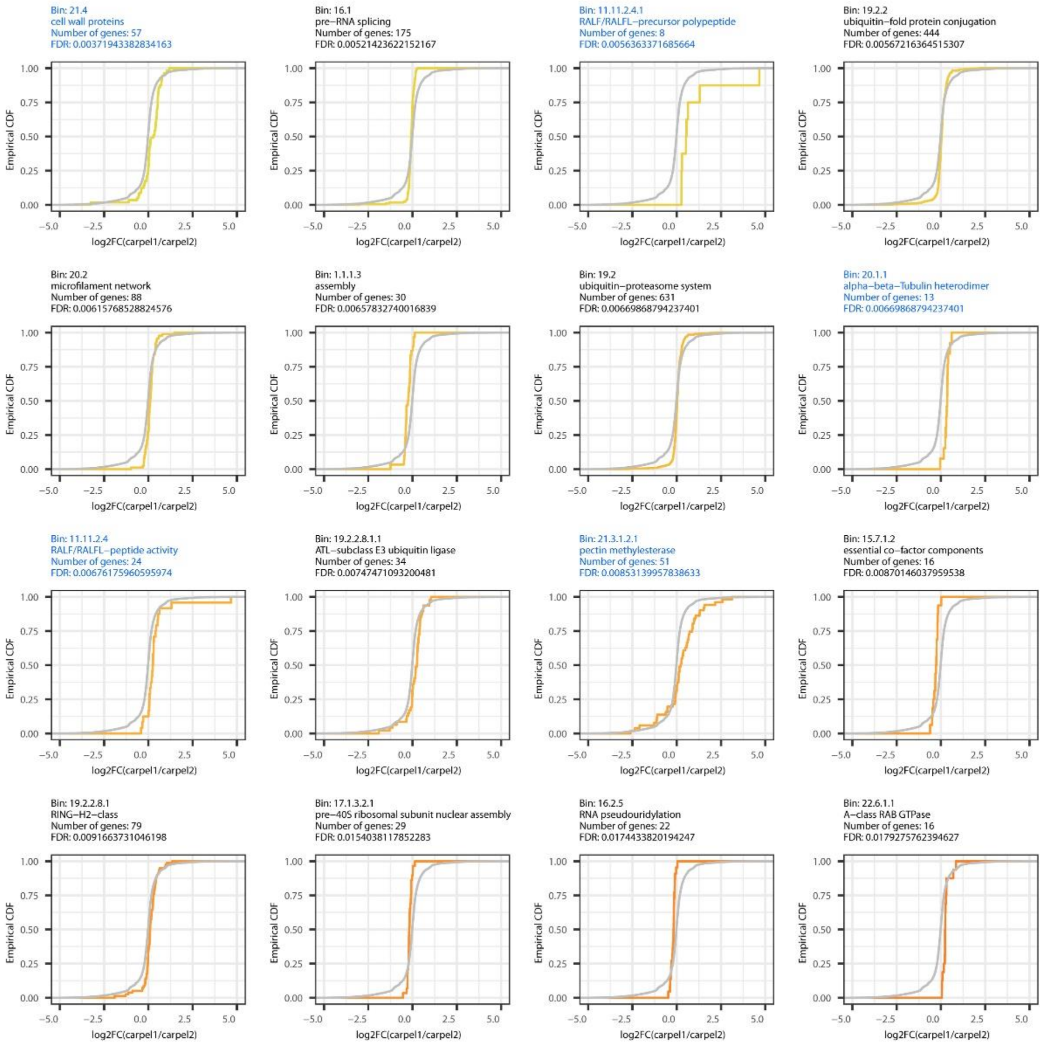
Bin: 21.4
cell wall proteins
Number of genes: 57
FDR: 0.00371943382834163
Bin: 16.1
pre−RNA splicing
Number of genes: 175
FDR: 0.00521423622152167
Bin: 11.11.2.4.1
RALF/RALFL−precursor polypeptide
Number of genes: 8
FDR: 0.0056363371685664
Bin: 19.2.2
ubiquitin−fold protein conjugation
Number of genes: 444
FDR: 0.00567216364515307
Bin: 20.2
microfilament network
Number of genes: 88
FDR: 0.00615768528824576
Bin: 1.1.1.3
assembly
Number of genes: 30
FDR: 0.00657832740016839
Bin: 19.2
ubiquitin−proteasome system
Number of genes: 631
FDR: 0.00669868794237401
Bin: 20.1.1
alpha−beta−Tubulin heterodimer
Number of genes: 13
FDR: 0.00669868794237401
Bin: 11.11.2.4
RALF/RALFL−peptide activity
Number of genes: 24
FDR: 0.00676175960595974
Bin: 19.2.2.8.1.1
ATL−subclass E3 ubiquitin ligase
Number of genes: 34
FDR: 0.00747471093200481
Bin: 21.3.1.2.1
pectin methylesterase
Number of genes: 51
FDR: 0.00853139957838633
Bin: 15.7.1.2
essential co−factor components
Number of genes: 16
FDR: 0.00870146037959538
Bin: 19.2.2.8.1
RING−H2−class
Number of genes: 79
FDR: 0.0091663731046198
Bin: 17.1.3.2.1
pre−40S ribosomal subunit nuclear assembly
Number of genes: 29
FDR: 0.0154038117852283
Bin: 16.2.5
RNA pseudouridylation
Number of genes: 22
FDR: 0.0174433820194247
Bin: 22.6.1.1
A−class RAB GTPase
Number of genes: 16
FDR: 0.0179275762394627
Empirical CDF
log2FC(carpel1/carpel2)
0.00
0.25
0.50
0.75
1.00
−5.0
−2.5
0.0
2.5
5.0

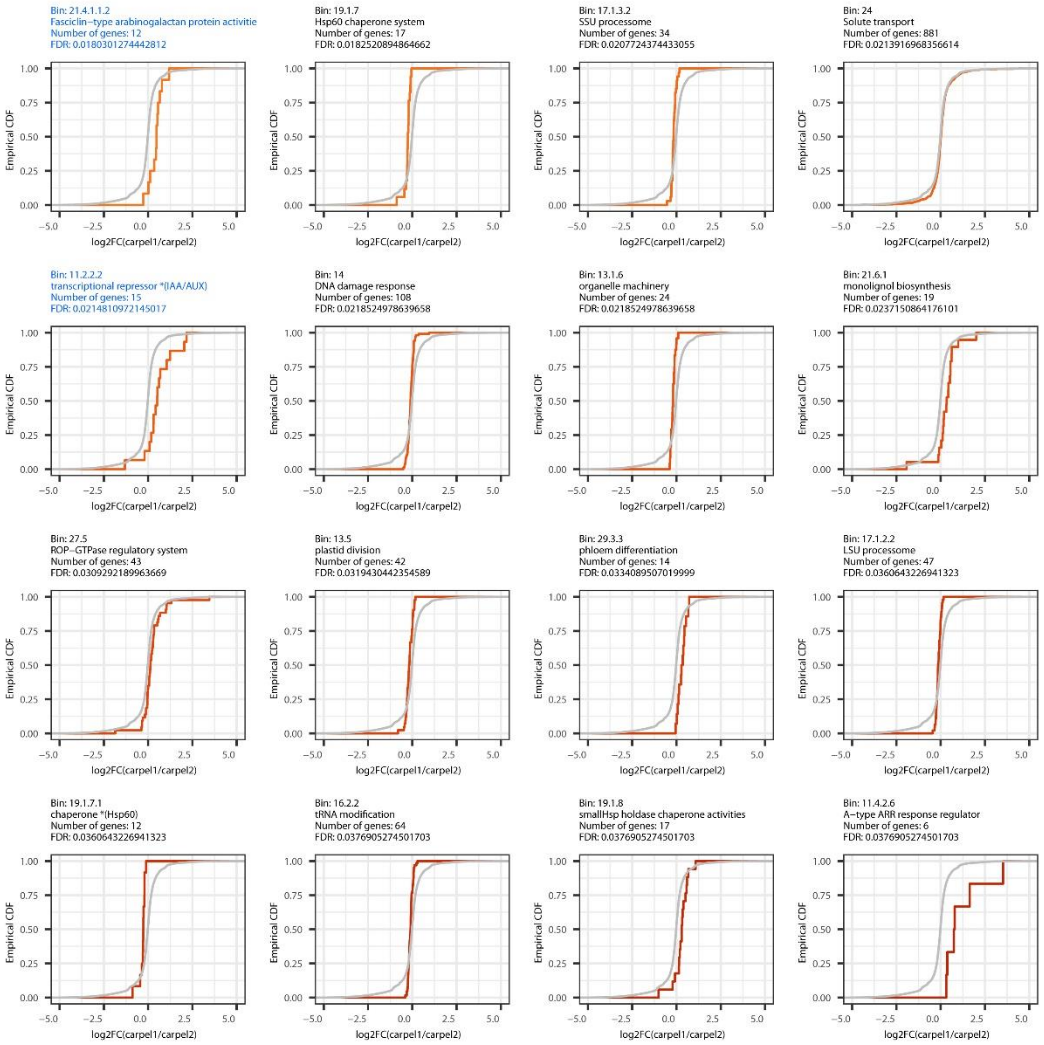
Bin: 21.4.1.1.2
Fasciclin–type arabinogalactan protein activitie
Number of genes: 12
FDR: 0.0180301274442812
Bin: 19.1.7
Hsp60 chaperone system
Number of genes: 17
FDR: 0.0182520894864662
Bin: 17.1.3.2
SSU processome
Number of genes: 34
FDR: 0.0207724374433055
Bin: 24
Solute transport
Number of genes: 881
FDR: 0.0213916968356614
Bin: 11.2.2.2
transcriptional repressor *(IAA/AUX)
Number of genes: 15
FDR: 0.0214810972145017
Bin: 14
DNA damage response
Number of genes: 108
FDR: 0.0218524978639658
Bin: 13.1.6
organelle machinery
Number of genes: 24
FDR: 0.0218524978639658
Bin: 21.6.1
monolignol biosynthesis
Number of genes: 19
FDR: 0.0237150864176101
Bin: 27.5
ROP–GTPase regulatory system
Number of genes: 43
FDR: 0.0309292189963669
Bin: 13.5
plastid division
Number of genes: 42
FDR: 0.0319430442354589
Bin: 29.3.3
phloem differentiation
Number of genes: 14
FDR: 0.0334089507019999
Bin: 17.1.2.2
LSU processome
Number of genes: 47
FDR: 0.0360643226941323
Bin: 19.1.7.1
chaperone *(Hsp60)
Number of genes: 12
FDR: 0.0360643226941323
Bin: 16.2.2
tRNA modification
Number of genes: 64
FDR: 0.0376905274501703
Bin: 19.1.8
smallHsp holdase chaperone activities
Number of genes: 17
FDR: 0.0376905274501703
Bin: 11.4.2.6
A–type ARR response regulator
Number of genes: 6
FDR: 0.0376905274501703
Empirical CDF
log2FC(carpel1/carpel2)

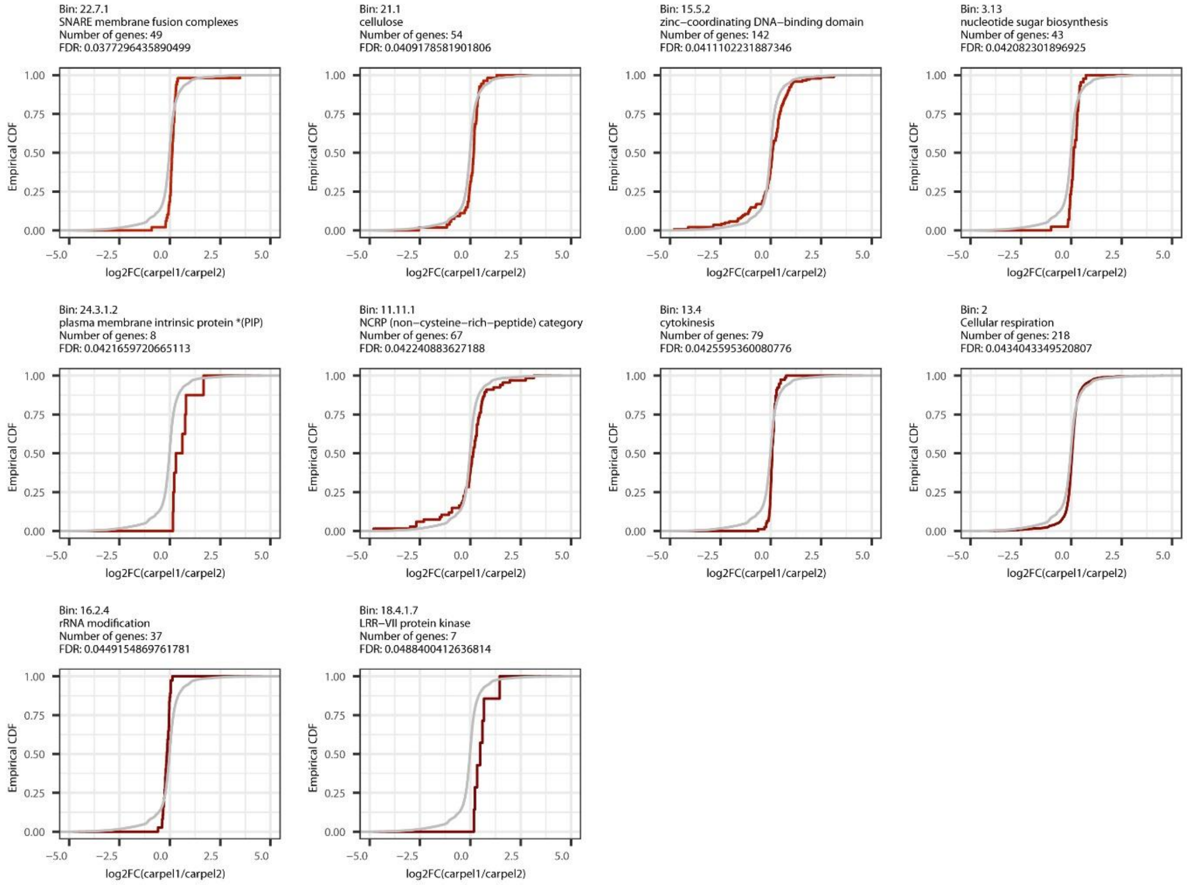
Bin: 22.7.1
SNARE membrane fusion complexes
Number of genes: 49
FDR: 0.0377296435890499
Bin: 21.1
cellulose
Number of genes: 54
FDR: 0.0409178581901806
Bin: 15.5.2
zinc–coordinating DNA–binding domain
Number of genes: 142
FDR: 0.0411102231887346
Bin: 3.13
nucleotide sugar biosynthesis
Number of genes: 43
FDR: 0.042082301896925
Bin: 24.3.1.2
plasma membrane intrinsic protein *(PIP)
Number of genes: 8
FDR: 0.0421659720665113
Bin: 11.11.1
NCRP (non–cysteine–rich–peptide) category
Number of genes: 67
FDR: 0.042240883627188
Bin: 13.4
cytokinesis
Number of genes: 79
FDR: 0.0425595360080776
Bin: 2
Cellular respiration
Number of genes: 218
FDR: 0.0434043349520807
Bin: 16.2.4
rRNA modification
Number of genes: 37
FDR: 0.0449154869761781
Bin: 18.4.1.7
LRR–VII protein kinase
Number of genes: 7
FDR: 0.0488400412636814
Empirical CDF
log2FC(carpel1/carpel2)

Figure S10: Mercator bins identified as having significantly different distributions of log2 fold-changes in expression between carpels 1 and 2 of *Cyanella alba* subsp. *flavescens*

ECDF plots are shown for all bins that were identified as significantly different from the transcriptomic background. For each bin, the bin number, short description, number of genes contained in the bin and FDR value is indicated, and the grey line is the transcriptomic background (all transcripts outside of the bin). Bins discussed in the main text are highlighted with blue font.

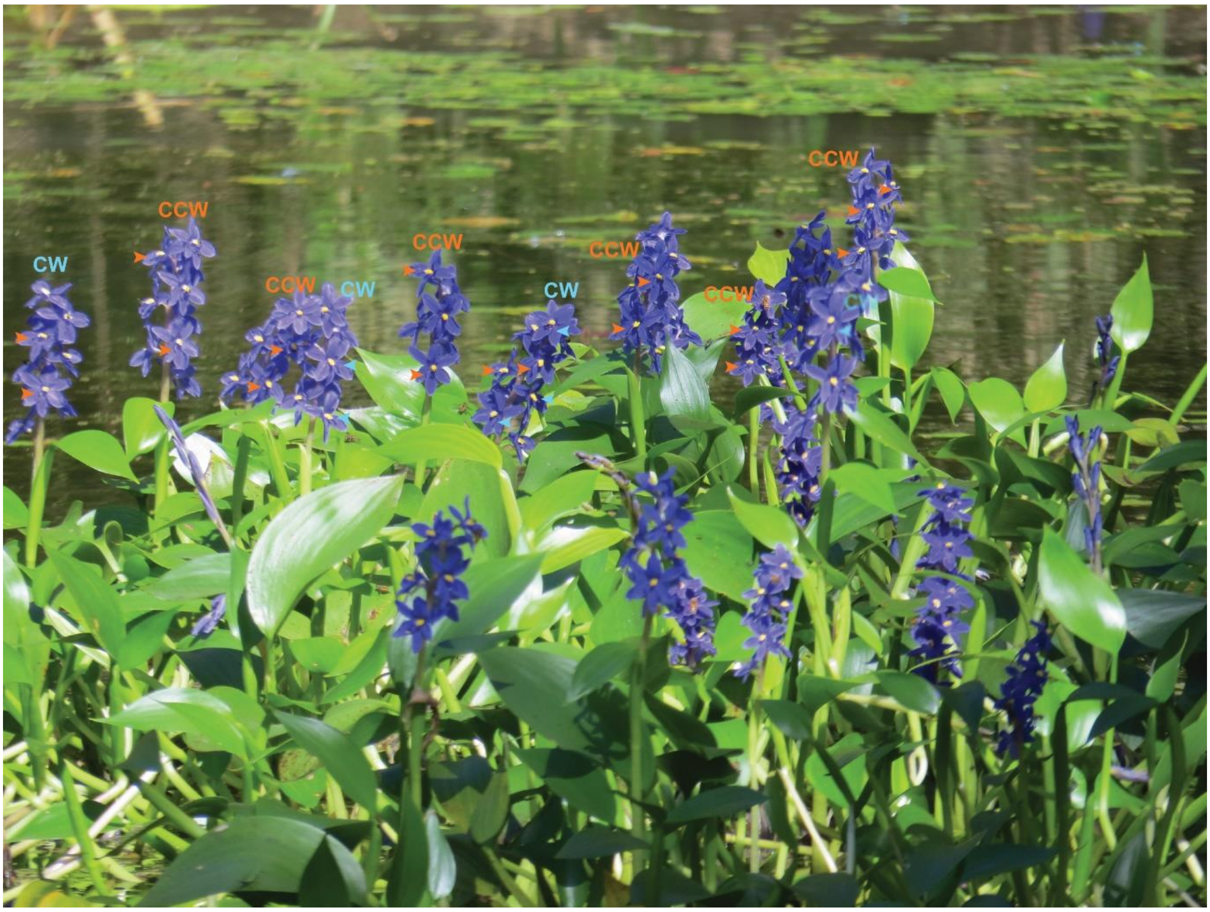

Figure S11: *Monochoria australasica* inflorescences, a species with inflorescence-level monomorphic enantiostyly

Phyllotaxis orientation is indicated as CW (clockwise) or CCW (counter clockwise). Orange and blue arrowheads point to styles deflected to the left (orange) or right (blue). See Table S9. Photo by Andrew C. Mitchell under https://www.inaturalist.org/observations/19364608 (license CC-BY).

Supporting Information Tables

Table S1: Numbers of 10-kb windows in the genome with different coverage between L- and R-plants in Illumina2021 and Illumina2022 datasets

In Haplotype 1 of the genome assembly		
	Windows with higher coverage in L-plants	Windows with higher coverage in R-plants
Illumina2021 dataset (25 individuals)	3	3
Illumina2022 dataset (20 individuals)	3	5
Shared by Illumina2021 and Illumina2022 datasets	0	0
Combined dataset (45 individuals)	2	1

In Haplotype 2 of the genome assembly		
	Windows with higher coverage in L-plants	Windows with higher coverage in R-plants
Illumina2021 dataset (25 individuals)	14	0
Illumina2022 dataset (20 individuals)	6	9
Shared by Illumina2021 and Illumina2022 datasets	0	0
Combined dataset (45 individuals)	4	3

Tables S2 to S8: See section on biomechanical model above in Supporting Information Text

Table S9: List of available *Monochoria austalasica* images with their phyllotaxis orientation and style deflection.

CW: clockwise, CCW: counterclockwise, when moving from older to younger organs.

Image link	Phyllotaxis	Style deflection
https://www.inaturalist.org/observations/74107844	CCW	L
https://www.inaturalist.org/observations/65301963	CW	R
https://www.inaturalist.org/observations/60589407	CCW	L
https://www.inaturalist.org/observations/40130455	CW	R
https://www.inaturalist.org/observations/19364608	CW	R
	CW	R
	CW	R
https://www.inaturalist.org/observations/19364608 (large image; see Figure S11)	CW	L
	CCW	L
	CCW	L
	CW	R
	CCW	L
	CW	mixed
	CCW	L
	CCW	L
	CCW	L
	CW	R
SCHB image	CCW	L
https://www.flickr.com/photos/71736071@N02/34452474370/	CCW	L
https://www.inaturalist.org/observations/151569011 (misidentified as *P. cyanea*)	CCW	L
https://www.inaturalist.org/observations/121835877 (misidentified as *P. cyanea*)	CCW	L
