# Supplementary material for: Spiral phyllotaxis predicts left-right asymmetric growth and style deflection in mirror-image flowers of Cyanella alba
Source: Nat Commun. 2025 Apr 18;16:3695. doi: 10.1038/s41467-025-58803-5 (PMC12008388; doi:10.1038/s41467-025-58803-5)
Supplement: Supplementary file 2 — Description of Additional Supplementary Files [file 41467_2025_58803_MOESM2_ESM.pdf]

Description of Additional Supplementary Files

Supplementary Data 1: List of genes upregulated in R- and L-buds

Supplementary Data 2: List of genes upregulated in carpel 1 and in carpel 2

Supplementary Data 3: Significant MapMan categories

Supplementary Movie S1: Time-lapse movie of style deflecting

Supplementary Software File lateR1.nb: Mathematica workbook for biomechanical model
