# Supplementary material for: Spiral phyllotaxis predicts left-right asymmetric growth and style deflection in mirror-image flowers of Cyanella alba
Source: Nat Commun. 2025 Apr 18;16:3695. doi: 10.1038/s41467-025-58803-5 (PMC12008388; doi:10.1038/s41467-025-58803-5)
Supplement: Supplementary file 8 — Reporting Summary [file 41467_2025_58803_MOESM8_ESM.pdf]

nature portfolio

Corresponding author(s): Michael Lenhard

Last updated by author(s): Mar 3, 2025

Reporting Summary

Nature Portfolio wishes to improve the reproducibility of the work that we publish. This form provides structure for consistency and transparency in reporting. For further information on Nature Portfolio policies, see our Editorial Policies and the Editorial Policy Checklist.

Statistics

For all statistical analyses, confirm that the following items are present in the figure legend, table legend, main text, or Methods section.

n/a	Confirmed	
☐	☒	The exact sample size (*n*) for each experimental group/condition, given as a discrete number and unit of measurement
☐	☒	A statement on whether measurements were taken from distinct samples or whether the same sample was measured repeatedly
☐	☒	The statistical test(s) used AND whether they are one- or two-sided<br>*Only common tests should be described solely by name; describe more complex techniques in the Methods section.*
☒	☐	A description of all covariates tested
☐	☒	A description of any assumptions or corrections, such as tests of normality and adjustment for multiple comparisons
☐	☒	A full description of the statistical parameters including central tendency (e.g. means) or other basic estimates (e.g. regression coefficient) AND variation (e.g. standard deviation) or associated estimates of uncertainty (e.g. confidence intervals)
☐	☒	For null hypothesis testing, the test statistic (e.g. *F*, *t*, *r*) with confidence intervals, effect sizes, degrees of freedom and *P* value noted<br>*Give P values as exact values whenever suitable.*
☒	☐	For Bayesian analysis, information on the choice of priors and Markov chain Monte Carlo settings
☒	☐	For hierarchical and complex designs, identification of the appropriate level for tests and full reporting of outcomes
☒	☐	Estimates of effect sizes (e.g. Cohen's *d*, Pearson's *r*), indicating how they were calculated

Our web collection on statistics for biologists contains articles on many of the points above.

Software and code

Policy information about availability of computer code

Data collection	Image analysis was performed using ImageJ (version 1.54a).
Data analysis	Statistical analysis was performed using R (version 4.3.0). The transcriptome analysis used Trinity version 2.14.0, Mercator4 v6.0, Salmon v1.9.0, STAR, BRAKER3 and OrthoDB v11. The association analysis used SAMtools version 1.3.1, BCFtools version 1.16, VCFtools version 0.1.16, PLINK version 1.9.0, mosdepth version 0.3.3, IGV version 2.14.1, and KmerGO2. The genome assembly used KMC version 3.2.1, GenomeScope2.0, hifiasm version 0.19.0-r534, BUSCO version 5.4.4 and QUAST version 5.2.0, Bedtools version 2.30.0.

For manuscripts utilizing custom algorithms or software that are central to the research but not yet described in published literature, software must be made available to editors and reviewers. We strongly encourage code deposition in a community repository (e.g. GitHub). See the Nature Portfolio guidelines for submitting code & software for further information.

Data

Policy information about availability of data

All manuscripts must include a data availability statement. This statement should provide the following information, where applicable:

- Accession codes, unique identifiers, or web links for publicly available datasets
- A description of any restrictions on data availability
- For clinical datasets or third party data, please ensure that the statement adheres to our policy

The whole-genome sequencing and transcriptome datasets generated in this work have been deposited in NCBI under accession code BioProject PRJNA1123744. All other data generated in this study are provided in the Source Data file.

Research involving human participants, their data, or biological material

Policy information about studies with human participants or human data. See also policy information about sex, gender (identity/presentation), and sexual orientation and race, ethnicity and racism.

Reporting on sex and gender	not applicable
Reporting on race, ethnicity, or other socially relevant groupings	not applicable
Population characteristics	not applicable
Recruitment	not applicable
Ethics oversight	not applicable

Note that full information on the approval of the study protocol must also be provided in the manuscript.

Field-specific reporting

Please select the one below that is the best fit for your research. If you are not sure, read the appropriate sections before making your selection.

☒ Life sciences ☐ Behavioural & social sciences ☐ Ecological, evolutionary & environmental sciences

For a reference copy of the document with all sections, see nature.com/documents/nr-reporting-summary-flat.pdf

Life sciences study design

All studies must disclose on these points even when the disclosure is negative.

Sample size	No prior-sample size calculation was performed. Sample sizes were chosen to be as large as logistically feasible. For transcriptomics the sample sizes correspond to the standard in the field.
Data exclusions	No data were excluded from the analyses.
Replication	The correlation between the direction of phyllotaxis and the direction of style deflection was confirmed by sampling in the 2023 and 2024 flowering season. The auxin effect on style deflection was shown to be reproducible based on two independent experiment. In the transcriptomic analysis of styles, each type of tissue had three biological replicates. In the transcriptomic analysis of carpel walls, each type of tissue had four biological replicates. For all other experiments, replicate numbers are given directly in the figures.
Randomization	Samples were randomly allocated to experimental groups.
Blinding	No blinding was performed.

Reporting for specific materials, systems and methods

We require information from authors about some types of materials, experimental systems and methods used in many studies. Here, indicate whether each material, system or method listed is relevant to your study. If you are not sure if a list item applies to your research, read the appropriate section before selecting a response.

Materials & experimental systems

n/a	Involved in the study
☒	☐ Antibodies
☒	☐ Eukaryotic cell lines
☒	☐ Palaeontology and archaeology
☒	☐ Animals and other organisms
☒	☐ Clinical data
☒	☐ Dual use research of concern
☐	☒ Plants

Methods

n/a	Involved in the study
☒	☐ ChIP-seq
☒	☐ Flow cytometry
☒	☐ MRI-based neuroimaging

Dual use research of concern

Policy information about dual use research of concern

Hazards

Could the accidental, deliberate or reckless misuse of agents or technologies generated in the work, or the application of information presented in the manuscript, pose a threat to:

No	Yes
☒	☐ Public health
☒	☐ National security
☒	☐ Crops and/or livestock
☒	☐ Ecosystems
☒	☐ Any other significant area

Experiments of concern

Does the work involve any of these experiments of concern:

No	Yes
☒	☐ Demonstrate how to render a vaccine ineffective
☒	☐ Confer resistance to therapeutically useful antibiotics or antiviral agents
☒	☐ Enhance the virulence of a pathogen or render a nonpathogen virulent
☒	☐ Increase transmissibility of a pathogen
☒	☐ Alter the host range of a pathogen
☒	☐ Enable evasion of diagnostic/detection modalities
☒	☐ Enable the weaponization of a biological agent or toxin
☒	☐ Any other potentially harmful combination of experiments and agents

Plants

Seed stocks	Only wild-grown plants of Cyanella alba flavescens were used, these were sampled from three populations in the Biedouw Valley (site 1 at 32°08'18" E19°10'57", 400 m a.s.l and site 2 at S32°11'25" E19°10'17", 560 m a.s.l.) and Wupperthal (site 3 at S32°16'17" E19° 12'42", 530 m a.s.l.).
Novel plant genotypes	not applicable
Authentication	not applicable
